# Sex-specific survival gene mutations are discovered as clinical predictors of clear cell renal cell carcinoma

**DOI:** 10.1038/s41598-024-66525-9

**Published:** 2024-07-09

**Authors:** Jia Hwang, Hye Eun Lee, Jin Seon Han, Moon Hyung Choi, Sung Hoo Hong, Sae Woong Kim, Ji Hoon Yang, Unsang Park, Eun Sun Jung, Yeong Jin Choi

**Affiliations:** 1 Department of Hospital Pathology, Seoul St. Mary’s Hospital, College of Medicine, The Catholic University of Korea, 222 Banpo-daero, Seocho-Gu, Seoul, 06591 Republic of Korea; 2 Department of Radiology, College of Medicine, Eunpyeong St. Mary’s Hospital, The Catholic University of Korea, Seoul, 03312 Republic of Korea; 3 Department of Urology, Seoul St. Mary’s Hospital, College of Medicine, The Catholic University of Korea, Seoul, 06591 Republic of Korea; 4 Department of Computer Science and Engineering, Sogang University, Seoul, 04107 Republic of Korea; 5 Department of Hospital Pathology, College of Medicine, Eunpyeong St. Mary’s Hospital, The Catholic University of Korea, Seoul, 03312 Republic of Korea

**Keywords:** Clear cell renal cell carcinoma, Sex, Gene, Mutation, NGS, Cancer genomics, Renal cell carcinoma, Tumour biomarkers

## Abstract

Although sex differences have been reported in patients with clear cell renal cell carcinoma (ccRCC), biological sex has not received clinical attention and genetic differences between sexes are poorly understood. This study aims to identify sex-specific gene mutations and explore their clinical significance in ccRCC. We used data from The Cancer Genome Atlas-Kidney Renal Clear Cell Carcinoma (TCGA-KIRC), The Renal Cell Cancer-European Union (RECA-EU) and Korean-KIRC. A total of 68 sex-related genes were selected from TCGA-KIRC through machine learning, and 23 sex-specific genes were identified through verification using the three databases. Survival differences according to sex were identified in nine genes (*ACSS3, ALG13, ASXL3, BAP1, JADE3, KDM5C, KDM6A, NCOR1P1*, and *ZNF449*). Female-specific survival differences were found in *BAP1* in overall survival (OS) (TCGA-KIRC, p = 0.004; RECA-EU, p = 0.002; and Korean-KIRC, p = 0.003) and disease-free survival (DFS) (TCGA-KIRC, p = 0.001 and Korean-KIRC, p = 0.000004), and *NCOR1P1* in DFS (TCGA-KIRC, p = 0.046 and RECA-EU, p = 0.00003). Male-specific survival differences were found in *ASXL3* (OS, p = 0.017 in TCGA-KIRC; and OS, p = 0.005 in RECA-EU) and *KDM5C* (OS, p = 0.009 in RECA-EU; and DFS, p = 0.016 in Korean-KIRC). These results suggest that biological sex may be an important predictor and sex-specific tailored treatment may improve patient care in ccRCC.

## Introduction

The incidence of clear cell renal cell carcinoma is twice as high in males as in females worldwide, and the prognosis is also worse in males^1–4^. Differences in subtypes^3^, prognosis^3^, and treatment response^5^ in ccRCC according to sex have been observed, but the underlying reasons are not well known^2,6^.

Sex-related differences in cancer have been reported independent of race, ethnicity, or geographic location^7,8^. According to the GLOBOCAN 2020 database^1^, increased overall cancer incidence rates (by 19%) and mortality rates (by 43%) in males have been reported worldwide. This pattern of increased cancer susceptibility in males has been observed not only in kidney cancer, but also in cancers of the bladder, lung, liver, stomach and other sites^1,2,7^. Differences in gene expression and mutation frequencies between males and females in cancer have been reported in various studies^2,5,7–10^. These findings have been observed in ccRCC^11^ in the USA^6,12^, Canada^13^, Europe^10,14,15^, and Asia^10^. However, the differences in genetic variation according to sex and clinical usefulness are not well studied. Proposed factors for the sex disparity include environmental factors, immunological^7^, hormonal^2,7^, genetic, and pharmacokinetic^16^ differences between males and females, X chromosome effects^7,8^, and differences in the efficiency of the immunological and genomic surveillance mechanisms between males and females^7^.

Despite the growing demand for personalized medicine for cancer treatment, biological sex has not received special attention in clinical practice, and differences in genetic variation between sexes are largely unknown. With the introduction of the concept of gender medicine to the field of oncology^16,17^, investigating and discovering sex-specific genetic variants is important as they can be used as biomarkers for personalized treatment^16,18^. Understanding sex differences and incorporating them into personalized treatment strategies, rather than relying on a one-size-fits-all approach for all ccRCC patients, is essential.

In this study, machine learning was performed on 417 ccRCC patients using The Cancer Genome Atlas-Kidney Renal Clear Cell Carcinoma (TCGA-KIRC) database. We identified 68 sex-related genes in ccRCC and analyzed their association with survival. In addition, we examined regional differences by comparing results from European patients (Renal Cell Cancer-European Union, RECA-EU, 422 patients) and Korean patients (Korea-KIRC, 120 patients).

## Methods

### Ethics approval and consent to participate

All procedures performed in this study were in accordance with the 1964 Helsinki declaration and its later amendments or comparable ethical standards and approved by the Institutional Review Board of Catholic University of Korea, Seoul St. Mary’s Hospital (approval no. 2018-2550-0008, date of approval: 20 November 2018). The retrospective genetic study and the treatment plan were conducted according to clinical guidelines and standard of care. The results of the current genetic study did not affect the treatment plan of patients following surgery. Informed written consent was obtained from all patients.

### Feature selection and machine learning for the discovery of sex-related genes

**Figure 1:**
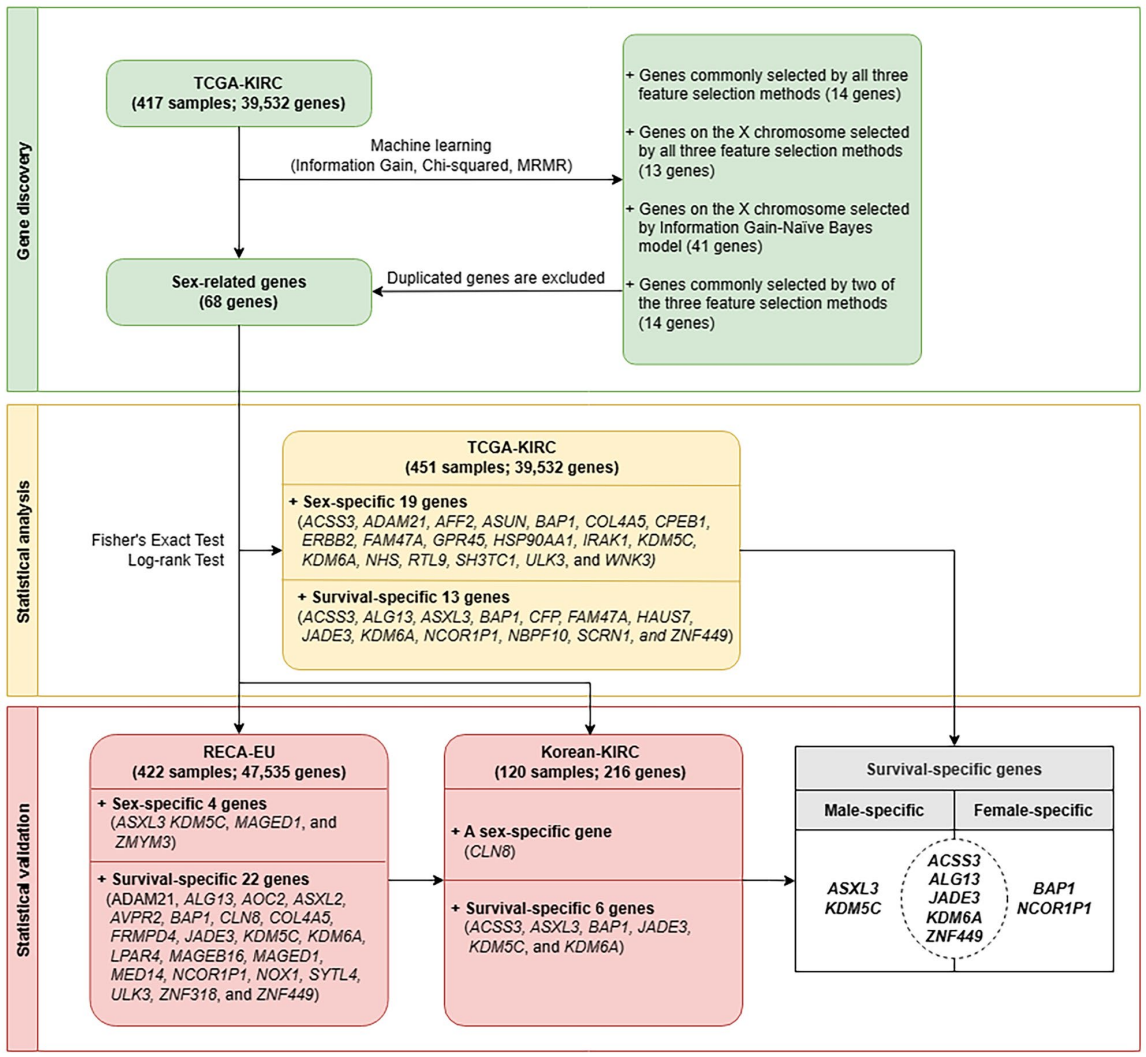
Workflow of the study. *ccRCC* clear cell renal cell carcinoma, *Korea-KIRC* Korea Kidney Renal Clear Cell Carcinoma, *MRMR* Minimum Redundancy and Maximum Relevance, *RECA-EU* Renal Cell Cancer-European Union, *TCGA-KIRC* The Cancer Genome Atlas-Kidney Renal Clear Cell Carcinoma.

The workflow of our study is shown in Fig. 1.

We first selected 417 patients with both somatic non-silent mutation data and clinical information from TCGA-KIRC (accessed on August 2016)^19^. The variant annotations of 39,532 genes of the 417 patients were obtained as an MAF file from UCSC Xena^20^. The machine learning methods utilized in this study were performed similarly to our previous study^21^. The cohort consists of 271 males and 146 females. We used Rapidminer (7.3 version, Boston, MA, USA) to implement data engineering and model building steps. The feature selection algorithm and classifiers used in the study are as follows: Information Gain, Chi-squared test, Minimum Redundancy Maximum Relevance (MRMR), Naïve Bayes, K-Nearest Neighbor (K-NN), and Support Vector Machine (SVM). The performances of the classification models in accordance with the feature selections were analyzed. We used tenfold cross validation for the model evaluation. Sex-related genes selected by machine learning were defined as genes showing differences in mutant rates based on sex.

### NGS-based ccRCC gene panel

A gene panel for ccRCC was designed using next-generation sequencing (NGS), consisting of 216 genes. The panel consists of 33 sex-related genes and 123 survival-specific genes, which were identified using machine learning in our research.^21^ Additionally, the panel includes 21 mutant genes with a mutation frequency above 5% in TCGA-KIRC and 14 genes associated with solid tumors, along with other 26 genes related with ccRCC.

### Targeted library preparation

Genomic DNA was extracted from formalin-fixed, paraffin-embedded (FFPE) tissues for library preparation. Genomic DNA was fragmented (approximately 250 bp fragments) using the Bioruptor Pico Sonication System (Diagenode, Belgium) and processed for Illumina sequencing by end-repair, dA-tailing, adapter ligation and pre-PCR for the indexed next generation sequencing (NGS) library. The prepared gDNA library and capture probes were hybridized to capture target regions using the Celemics target enrichment kit (Celemics, Seoul, Republic of Korea). Customized capture probes were designed and chemically synthesized to hybridize the target region. Captured regions were further amplified by post-PCR to enrich the amount of sample. The target-captured library was then sequenced on an Illumina NextSeq550 instrument (Illumina, San Diego, CA, USA) using the read layout 2 × 150 bp. The sequencing coverage and quality statistics for each sample are summarized in Additional file 1.

### Bioinformatics analysis

Samples were sequenced by the Nextseq 550 platform, Illumina Inc. BCL2FASTQ version 2.19.1.403 (Illumina) was used to demultiplex the base-call image files into individual sequence read files (FASTQ format). All options and parameters followed default settings. Sequencing adapters were removed by AdapterRemoval version 2. 2. 2.^22^, after low quality bases were removed by in-house code. All sequencing reads were aligned to the GRCh37 human genome by BWA-MEM (Burrows-Wheeler Aligner) software. The program uses the Burrows–Wheeler Transform algorithm to index the human genome sequence for calculating the constant complexity of each sequencing read. Post-align and recalibration processes were performed by Picard version 1.115 (http://broadinstitute.github.io/picard) and GATK^23^ version 4.0.4.0. We performed variant calling with GATK Haplotype caller. All detailed parameters and options followed best practices.

### Datasets

For validation of the identified sex-related genes in ccRCC, two publicly available and two private datasets were used. The TCGA-KIRC (accessed on 7 April 2021)^19^ and RECA-EU (https://dcc.icgc.org/projects/RECA-EU; accessed on 27 May 2021) datasets provide variants and clinical information of patients with RCC. Gene sequencing data and clinical information were available for 451 and 422 patients, respectively, including 293 males and 158 females in the TCGA-KIRC dataset (Additional file 2) and 245 males and 177 females in the RECA-EU dataset (Additional file 3).

Under the approval of the National Biobank of Korea, the Centers for Disease Control and Prevention, Republic of Korea, we acquired data from the Korean Chip, which contains genomic sequencing reads of a normal population in South Korea, from the Korea Biobank Array Project (KBN-2019-019, approval date: 21 March 2019). The project was initiated in 2014 by the Korea National Institute of Health and included 210,000 participants aged 40–69 years from the Korean Genome and Epidemiology Study^24^; these data were used for a customized Korean genome structure-based array with high genomic coverage and abundant functional variants of low to rare frequency^25^. The Korean Chip covers more than 833,000 markers including approximately 247,000 rare-frequency or functional variants estimated from approximately 2500 sequencing data in Koreans. Of the 833,000 markers, 208,000 functional markers were genotyped. More than 89,000 markers are present in East Asians.

For further validation, we chose 120 Korean patients diagnosed with ccRCC (Korean-KIRC) through either radical or partial nephrectomy and pathologic examination at The Catholic University of Korea, Seoul, St. Mary’s Hospital (Additional files 4 and 5). All participants provided signed informed consent for participation in the study. The cohort included 79 males and 41 females who underwent surgical treatment. Kidney samples were prepared from FFPE tissue and included 59 normal-tumor pairs and 61 tumor-only samples.

### Data pre-processing

A variant call format file store gene sequence variations was processed using PLINK 1.9 (www.cog-genomics.org/plink/1.9; accessed on 15 November 2021). Single nucleotide polymorphisms were genotyped to genomic variants using Ensembl Variant Effect Predictor (Version 96, http://apr2019.archive.ensembl.org/index.html; accessed on 15 November 2021), and identifiers for gene annotation were added using the biomaRt (https://bioconductor.org/packages/release/bioc/html/biomaRt.html) package for R (version 4.3.1, https://www.r-project.org/)^26^. To focus on the presence of cancerous mutations in ccRCC, variants that were also identified in normal tissues were removed. Python (version 3.11.4, https://www.python.org/) was used to preprocess raw data. Non-synonymous mutations were only considered in this study with eliminating synonymous and intron variants. Variants with less than 2% of variant allele frequency, less than five alternate allele count and less than 100 reading depth were excluded. Finally, the benign and likely benign variants were discarded as determined by clinical significance of variants with reference to the ClinVar^27^.

### Gene set enrichment analysis

Gene set enrichment analysis (GSEA) was performed using the Enrichr server^28^ (https://maayanlab.cloud/Enrichr/) to find out the biological processes and molecular function of sex-specific genes discovered from TCGA-KIRP and Korean-KIRP databases. This involved performing Kyoto Encyclopedia of Genes and Genomes (KEGG) 2021 Human and Gene Ontology (GO) Biological Process 2021 databases.

### Statistical analysis

To examine associations between sex and mutations, the Fisher’s Exact test was performed with the stats module of the Scipy package (version 1.8.1), Python (version 3.11.4) which deduces the sex-specific genes showing statistical specificity in mutation frequency based on sex. The survival probabilities in male and female patients were estimated by the Kaplan–Meier method and the Log-Rank Test using Python library called lifelines^29^. Statistical significance was determined with p < 0.05 as a threshold and with a 95% confidence level. The Fisher's exact test was performed using the R package (stats version 0.1.0).

## Results

### Machine learning was performed and 68 sex-related genes were selected from TCGA-KIRC

The TCGA-KIRC cohort consisted of 451 ccRCC patients; 293 (65%) were male and 158 (35%) were female (Supplementary Table S1). Machine learning was used to select sex-related genes from TCGA-KIRC. We evaluated the accuracy of classification algorithms including Naïve Bayes, K-NN, and SVM, and the best performing classifier was Naïve Bayes. Among the three feature selection methods, Naïve Bayes showed the highest accuracy of 98.80% (931 genes) when used with Information Gain (Supplementary Table S2). The classification prediction accuracy using other classification algorithms combined with feature selection methods are summarized in Supplementary Table S3.

A total of 68 sex-related genes from the TCGA-KIRC database were selected through four different methods of machine learning. First, the top 100 genes that were weighted and ranked by each feature selection method (Information gain, Chi-squared, MRMR) were selected, and the 14 genes commonly selected by all three feature selection methods were extracted (Supplementary Table S4; Supplementary Fig. S1). Second, 13 genes located on the X chromosome were discovered among the top 100 genes selected by three feature selection (Supplementary Table S5; Supplementary Fig. S2). Third, 41 genes located on the X chromosome of 931sex-related genes were discovered (Supplementary Table S6a,b). Finally, 14 genes commonly selected by two feature selection methods were extracted among the top 100 genes ranked by each feature selection (Supplementary Table S7). Among the top 20 genes related with sex that were extracted by each feature selection method, *BAP1* was extracted as the top gene in all methods and predicted to be the most important sex-related gene in ccRCC (Table 1).

**Table 1:** Top 20 sex-related genes identified by feature selection methods in the TCGA-KIRC database.

	Information gain weight		Chi-square weight		MRMR weight	
Top 10	BAP1**	0.02	BAP1**	12.3	BAP1**	1
	KDM5C*	0.015	PALM2-AKAP2*	8.04	*ITGAX*	1
	ASXL2**	0.015	ASXL2**	7.5	CFP**	1
	AFF2*	0.015	AFF2*	7.5	*C11orf30*	1
	KRTAP1-1*	0.015	KRTAP1-1*	7.5	*SLC17A2*	1
	ADAM21**	0.015	ADAM21**	7.5	*TTC29*	1
	SCAF1*	0.015	SCAF1*	7.5	*ACSS3*	1
	ERBB2**	0.015	ERBB2**	7.5	ASXL2**	1
	LRP12**	0.015	LRP12**	7.5	*ZFP42*	1
	AGK*	0.015	AGK*	7.5	*POMT2*	1
Top 20	DYSF*	0.015	DYSF*	7.5	*ZNF318*	1
	HSP90AA1*	0.015	HSP90AA1*	7.5	*ULK3*	1
	NBPF10**	0.015	NBPF10**	7.5	*ZNFX1*	1
	SUCLG1*	0.015	SUCLG1*	7.5	*AOC2*	1
	PALM2-AKAP2*	0.014	KDM5C*	7.25	ADAM21**	1
	*ASXL3*	0.012	*MAP3K15*	6.25	*CLN8*	1
	*TET2*	0.012	*COL22A1*	6.25	ERBB2**	1
	*PABPC3*	0.012	*TGM5*	6.25	*MLLT3*	1
	CPEB1*	0.011	CPEB1*	5.61	LRP12**	1
	CFP**	0.011	CFP**	5.61	NBPF10**	1

*Is used for genes commonly selected by two types of feature selection. **Are used for genes commonly selected by all three types of feature selection. *MRMR* Minimum Redundancy-Maximum Relevance, *TCGA-KIRC* The Cancer Genome Atlas-Kidney Renal Clear Cell Carcinoma.

### Twenty-three sex-specific genes were verified by statistical analysis in TCGA-KIRC, RECA-EU and Korean-KIRC databases

The 68 sex-related genes were verified statistically with the RECA-EU and Korean-KIRC databases, and total 23 sex-specific genes were identified (Table 2).

**Table 2:** Comparison of 23 sex-specific genes from 68 sex-related genes in TCGA-KIRC, RECA-EU and Korean-KIRC databases.

No	Database	Gene	TCGA-KIRC				RECA-EU				Korean-KIRC			
			Mutation frequency (%)				Mutation frequency (%)				Mutation frequency (%)			
			Total	Male	Female	*p*-Value	Total	Male	Female	*p*-Value	Total	Male	Female	*p*-Value
1	TCGA-KIRC	*ACSS3*	0.67	0	1.90	0.042*	2.61	2.45	2.82	1.000	2.50	3.80	0	0.550
2		*ADAM21*	0.89	0	2.53	0.015*	1.18	1.22	1.13	1.000	0	0	0	–
3		*AFF2*	1.55	0.34	3.8	0.009*	0.71	0.82	2.26	0.243	2.04	3.17	0	0.538
4		*ASUN*	0.67	0	1.90	0.042*	–	–	–	–	0	0	0	–
5		*BAP1*	8.87	5.80	14.56	0.003*	13.27	12.24	14.69	0.471	3.33	2.53	4.88	0.605
6		*COL4A5*	2.00	0.68	4.43	0.011*	2.37	1.63	3.39	0.333	62.24	66.67	54.29	0.274
7		*CPEB1*	0.67	0	1.90	0.042*	0.95	1.63	0	0.143	0	0	0	–
8		*ERBB2*	0.89	0	2.53	0.015*	2.61	2.86	2.26	0.767	55.00	55.70	53.66	0.849
9		*FAM47A*	0.89	0	2.53	0.015*	0.71	0.41	1.13	0.575	4.17	5.06	2.44	0.660
10		*GPR45*	0.67	0	1.90	0.042*	0.47	0.41	0.56	1.000	0	0	0	–
11		*HSP90AA1*	0.89	0	2.53	0.015*	1.42	1.63	1.13	1.000	0	0	0	–
12		*IRAK1*	0.67	0	1.90	0.042*	0.47	0.82	0	0.512	0	0	0	–
13		*KDM5C*	5.99	7.85	2.53	0.023*	10.19	13.47	5.65	0.009*	0.83	1.27	0	1.000
14		*KDM6A*	0.67	0	1.90	0.042*	2.84	1.63	4.52	0.134	0.83	1.27	0	1.000
15		*NHS*	0.67	0	1.90	0.042*	1.42	0.82	2.26	0.243	55.10	53.97	57.14	0.830
16		*RTL9*	0.89	0	2.53	0.015*	–	–	–	–	0	0	0	–
17		*SH3TC1*	0.67	0	1.90	0.042*	4.03	3.67	4.52	0.803	0	0	0	–
18		*ULK3*	0.67	0	1.90	0.042*	1.18	0.82	1.69	0.654	0	0	0	–
19		*WNK3*	0.67	0	1.90	0.042*	1.18	0.41	2.26	0.166	0	0	0	–
20	RECA-EU	*ASXL3*	1.77	2.73	0	0.055	1.90	3.27	0	0.023*	9.18	11.11	5.71	0.485
21		*MAGED1*	0.44	0.68	0	0.544	0.95	0	2.26	0.030*	–	–	–	–
22		*ZMYM3*	0.67	0.34	1.27	0.282	1.18	0	2.82	0.013*	0	0	0	–
23	Korean-KIRC	*CLN8*	0.44	0	1.27	0.122	0.71	0.41	1.13	0.575	9.18	14.29	0	0.024*

*Is used for *p*-values < 0.05. Statistical significant values are bolded. The *p*-values are from the Fisher’s exact test. *Korean-KIRC* Korean-Kidney Renal Clear Cell Carcinoma, *RECA-EU* Renal Cell Cancer-European Union, *TCGA-KIRC* The Cancer Genome Atlas-Kidney Renal Clear Cell Carcinoma.

We identified 19 sex-specific genes from the 68 sex-related genes by statistical analysis in TCGA-KIRC (Supplementary Table S8). All genes are frequently or only mutated in females except for *KDM5C* (Supplementary Fig. S3). *KDM5C* was the only gene mutated predominantly in males [male:female = 7.85:2.53 (odds ratio = 2.74; p = 0.023). Among the 19 sex-specific genes, nine genes were located on the X chromosome (*AFF2, COL4A5, FAM47A, IRAK1, KDM5C, KDM6A, NHS, RTL9*, and *WNK3*). Four genes (*ASXL3*, *KDM5C*, *MAGED1* and *ZMYM3*) were verified as sex-specific in the RECA-EU data (Supplementary Table S9). *ASXL3* mutation occurred only in males in both TCGA-KIRC (2.73%, p = 0.055) and RECA-EU (3.27%, p = 0.023), and was also found at a higher frequency in males in Korean-KIRC [male:female = 11.11:5.71 (p = 0.485)]. *KDM5C* mutations also occurred more frequently in males [male:female = 13.47:5.65 in RECA-EU (odds ratio = 2.43; p = 0.009)]. A total of 216 genes were verified in the Korean-KIRC database. We also investigated the mutation frequencies of the 68 sex-related genes in ccRCC using the Korean Chip, which provides genome sequencing reads of 210,000 healthy Koreans^25^. Mutations of the 68 genes were not found in Koreans without ccRCC. Sex-specifically verified among the 33 sex-related genes in Korean-KIRC was exclusively *CLN8*. (Supplementary Table S10). *CLN8* mutations were found only in females (1.27%) in TCGA-KIRC, more frequently in females (1.13%) than males (0.41%) in RECA-EU. However, in Korean-KIRC, *CLN8* mutations were identified solely in males (14.29%, odds ratio = 6.39; p = 0.024).

### Survival analysis of 68 sex-related genes and 23 sex-specific genes using three databases

We conducted survival analysis on the 68 sex-related genes using the TCGA-KIRC, RECA-EU and Korean-KIRC databases. In TCGA-KIRC, *ASXL3*, *HAUS7*, and *NBPF10* were survival-specific only for males in OS (p = 0.017, p = 0.042, and p = 0.008). However, *ACSS3, ALG13, BAP1, CFP, FAM47A, JADE3, KDM6A, NCOR1P1, SCRN1,* and *ZNF449* were survival-specific only in females. The data for these genes are presented in Supplementary Table S11. Collective survival graphs depending on the presence of mutations in the male-specific survival genes (*ASXL3, HAUS7,* and *NBPF10*) and the female-dependent survival genes in OS and DFS (*ACSS3, BAP1, CFP,* and *FAM47A*) of TCGA-KIRC were shown in Supplementary Fig. S5. Survival-specific genes showing sex differences in the RECA-EU and Korean-KIRC databases can be found in Supplementary Tables S12 and S13, respectively.

Differences in survival analysis by sex for 23 sex-specific genes were compared among the three databases (Table 3).

**Table 3:** Comparative survival analysis for 23 sex-specific genes in the TCGA-KIRC, RECA-EU and Korean-KIRC databases.

**#**	Database	Gene	TCGA-KIRC						RECA-EU						Korean-KIRC					
			Overall survival			Disease-free survival			Overall survival			Disease-free survival			Overall survival			Disease-free survival		
			Total	Male	Female	Total	Male	Female	Total	Male	Female	Total	Male	Female	Total	Male	Female	Total	Male	Female
1	TCGA-KIRC	*ACSS3*	0.0002**	–	0.0001**	0.003*	–	0.002*	0.522	0.494	0.877	0.708	0.813	0.774	0.416	0.368	–	0.016*	0.026*	–
2		*ADAM21*	0.246	–	0.277	0.402	–	0.430	0.074	0.033*	0.577	0.820	0.894	0.840	–	–	–	–	–	–
3		*AFF2*	0.407	0.695	0.340	0.370	0.640	0.168	0.667	0.436	0.824	0.792	0.893	0.805	0.836	0.855	–	0.441	0.441	–
4		*ASUN*	0.357	–	0.490	0.691	–	0.539	–	–	–	–	–	–	–	–	–	–	–	–
5		*BAP1*	0.052	0.662	0.004*	**0.025**	0.670	0.001*	0.002*	0.202	0.002*	0.409	0.607	0.519	0.234	0.773	0.003*	0.00002**	0.222	0.000004**
6		*COL4A5*	0.741	0.370	0.407	0.592	0.321	0.708	0.331	0.026*	0.721	0.732	0.858	0.748	0.282	0.490	0.374	0.515	0.515	0.668
7		*CPEB1*	0.691	–	0.795	0.740	–	0.666	0.830	0.764	–	0.814	0.848	–	–	–	–	–	–	–
8		*ERBB2*	0.177	–	0.158	0.196	–	0.235	0.133	0.299	0.258	0.694	0.798	0.774	0.824	0.743	0.930	0.427	0.558	0.723
9		*FAM47A*	0.004*	–	0.010*	0.000007**	–	0.000003**	0.486	0.828	0.538	0.891	0.948	0.887	0.506	0.525	0.822	0.995	0.864	0.714
10		*GPR45*	0.999	–	0.967	0.848	–	0.726	0.542	0.525	1.000	0.907	0.924	1.000	–	–	–	–	–	–
11		*HSP90AA1*	0.421	–	0.522	0.372	–	0.409	0.759	0.877	0.470	0.789	0.858	0.840	–	–	–	–	–	–
12		*IRAK1*	0.212	–	0.238	0.462	–	0.381	0.339	0.393	–	0.899	0.908	–	–	–	–	–	–	–
13		*KDM5C*	0.145	0.070	0.869	0.166	0.360	0.268	0.023*	0.009*	0.838	0.476	0.596	0.681	0.080	0.107	–	0.011*	0.016*	–
14		*KDM6A*	0.017*	–	0.032*		–	–	0.016*	0.042*	0.096	0.019*	0.848	0.012*	0.853	0.872	–	0.035*	0.049*	–
15		*NHS*	0.707	–	0.762	0.741	–	0.673	0.192	0.403	0.339	0.792	0.893	0.805	0.864	0.270	0.403	0.973	0.973	0.802
16		*RTL9*	0.249	–	0.236	0.254	–	0.294	–	–	–	–	–	–	–	–	–	–	–	–
17		*SH3TC1*	0.176	–	0.163	0.908	–	0.948	0.349	0.463	0.506	0.659	0.807	0.681	–	–	–	–	–	–
18		*ULK3*	0.062	–	0.080	0.487	–	0.355	0.0001**	0.00004**	0.037*	0.835	0.908	0.840	–	–	–	–	–	–
19		*WNK3*	0.894	–	0.973	0.391	–	0.430	0.652	0.500	0.994	0.810	0.924	0.805	–	–	–	–	–	–
20	RECA-EU	*ASXL3*	0.022*	0.017*	–	0.780	0.697	–	0.617	0.693	–	0.752	0.791	–	0.050*	0.005*	0.803	0.090	0.090	0.656
21		*MAGED1*	0.394	0.411	–	0.390	0.377	–	0.312	–	0.323	**6.04E**–07**	–	0.00003**	–	–	–	–	–	–
22		*ZMYM3*	0.967	0.657	0.889	0.408	0.598	0.573	0.614	–	0.494	0.792	–	0.748	–	–	–	–	–	–
23	Korean-KIRC	*CLN8*	0.417	–	0.401	0.416	–	0.459	0.588	0.025*	0.532	0.863	0.924	0.871	0.648	0.680	–	0.777	0.777	–

*Is used for *p*-values < 0.05. **Are used for *p*-values < 0.001. Statistical significant values are bolded. The *p*-values are from the Log-rank test. *Korean-KIRC* Korean-Kidney Renal Clear Cell Carcinoma, *RECA-EU* Renal Cell Cancer-European Union, *TCGA-KIRC* The Cancer Genome Atlas-Kidney Renal Clear Cell Carcinoma.

In TCGA-KIRC, four genes (*ACSS3*, *BAP1*, *FAM47A*, *and KDM6A)* were survival-specific only for females in overall survival (OS) (p = 0.0001, p = 0.004, p = 0.010,and p = 0.032, respectively) and disease-free survival (DFS) (p = 0.002, p = 0.001, p = 0.000003, and p = NA, respectively). Individual survival graphs of male and female patients with mutations in the four sex-specific and survival-specific genes from TCGA-KIRC were shown in Supplementary Fig. S4. *ASXL3* mutations were significantly correlated with OS (p = 0.017) only in males. As a result of survival analysis, 8 genes (*ADAM21, BAP1, COL4A5, KDM5C, KDM6A, ULK3, MAGED1,* and *CLN8*) among 23 sex-specific genes in the RECA-EU dataset showed survival-specific significance. Male-specific survival differences were found in *ADAM21, COL4A5, KDM5C*, and *CLN8* (p = 0.033, p = 0.026, p = 0.009, and p = 0.025, respectively) in OS. *BAP1* and *MAGED1* were female-specific in OS (p = 0.002) and DFS (p = 0.00003). *KDM6A* was male-specific in OS (p = 0.042), whereas female-specific in DFS (p = 0.012). The *ULK3* gene mutation was specific in OS for both males and females (p = 0.002 and p = 0.037). In the Korean-KIRC database, a total of 5 genes (*ACSS3*, *BAP1*, *KDM5C*, *KDM6A*, and *ASXL3*) were identified as survival-specific among the 23 sex-specific genes. *BAP1* was female-specific in both OS (p = 0.003) and DFS (p = 0.000004). On the other hand, *ACSS3*, *KDM5C*, and *KDM6A* were male-specific in DFS (p = 0.026, p = 0.016, and p = 0.049), while *ASXL3* was also male-specific in OS (p = 0.005).

### Nine survival genes showing sex differences were compared between three databases

Combining the above results, nine survival-specific genes (*ACSS3, ALG13, ASXL3, BAP1, JADE3, KDM5C, KDM6A, NCOR1P1,* and *ZNF449*) that were commonly identified in two or more databases were analyzed according to sex (Table 4).

**Table 4:** Sex-specific survival differences in 9 survival-specific genes commonly identified in TCGA-KIRC, RECA-EU and Korean-KIRC databases.

No	Gene	Database	Mutation frequency (%)				Overall survival			Disease-free survival			Sex specificity in survival
			Total	Male	Female	*p*-Value	Total	Male	Female	Total	Male	Female	
1	*ACSS3*^§^	TCGA-KIRC	0.67	0	1.90	0.042*	0.0002**	–	0.0001**	0.003*	–	0.002*	Female
		RECA-EU	2.61	2.45	2.82	1.000	0.522	0.494	0.877	0.708	0.813	0.774	–
		Korean-KIRP	2.50	3.80	0	0.550	0.416	0.368	–	0.016*	0.026*	–	Male
2	*ALG13*^§^	TCGA-KIRC	0.89	0.34	1.90	0.126	0.060	0.829	0.018*	0.619	0.755	0.693	Female
		RECA-EU	1.66	1.63	1.69	1.000	0.080	0.010*	0.874	0.0006**	0.881	0.0001**	Male/female
		Korean-KIRP	0	0	0	–	–	–	–	–	–	–	–
3	*ASXL3*^§^	TCGA-KIRC	1.77	2.73	0	0.055	0.022*	0.017*	–	0.780	0.697	–	Male
		RECA-EU	1.90	3.27	0	0.023*	0.617	0.693	–	0.752	0.791	–	–
		Korean-KIRP	9.18	11.11	5.71	0.485	0.050*	0.005*	0.803	0.172	0.090	0.656	Male
4	*BAP1*^†^	TCGA-KIRC	8.87	5.80	14.56	0.003*	0.052	0.662	0.004*	0.025*	0.670	0.001*	Female
		RECA-EU	13.27	12.24	14.69	0.471	0.002*	0.202	0.002*	0.409	0.607	0.519	Female
		Korean-KIRP	3.33	2.53	4.88	0.605	0.234	0.773	0.003*	0.00002**	0.222	0.000004**	Female
5	*JADE3*^†^	TCGA-KIRC	0.44	0	1.27	0.122	0.019*	–	0.046*	–	–	–	Female
		RECA-EU	0.24	0.41	0	1.000	1.81E−92**	3.93E−54**	–	1.000	1.000	–	Male
		Korean-KIRP	0.83	1.27	0	1.000	0.00008**	0.00005**	–	0.0003**	0.00002**	–	Male
6	*KDM5C*^§^	TCGA-KIRC	5.99	7.85	2.53	0.023*	0.145	0.070	0.869	0.166	0.360	0.268	–
		RECA-EU	10.19	13.47	5.65	0.009*	0.023*	0.009*	0.838	0.476	0.596	0.681	Male
		Korean-KIRP	0.83	1.27	0	1.000	0.080	0.107	–	0.011*	0.016*	–	Male
7	*KDM6A*^†^	TCGA-KIRC	0.67	0	1.90	0.042*	0.017*	–	0.032*	–	–	–	Female
		RECA-EU	2.84	1.63	4.52	0.134	0.016*	0.042*	0.096	0.019*	0.848	0.012*	Male/female
		Korean-KIRP	0.83	1.27	0	1.000	0.853	0.872	–	0.035*	0.049*	–	Male
8	*NCOR1P1*^§^	TCGA-KIRC	1.55	0.68	3.16	0.054	0.472	0.335	0.055	0.714	0.300	0.046*	Female
		RECA-EU	1.66	1.63	1.69	1.000	0.418	0.248	0.859	0.002*	0.848	0.00003**	Female
		Korean-KIRP	–	–	–	–	–	–	–	–	–	–	–
9	*ZNF449*^§^	TCGA-KIRC	0.89	0.68	1.27	0.615	0.078	0.425	0.012*	0.494	0.558	0.062	Female
		RECA-EU	0.72	0.41	1.13	0.575	0.009*	0.001*	0.169	0.891	0.948	0.887	Male
		Korean-KIRP	0	0	0	–	–	–	–	–	–	–	–

^†^Is used for genes that are survival-specific in all three databases. ^§^Is used for genes that are survival-specific in two databases. *Is used for *p*-values < 0.05. **Are used for *p*-values < 0.001. Statistical significant values are bolded. The *p*-values are from the Fisher’s exact test and Log-rank test. *Korean-KIRC* Korean-Kidney Renal Clear Cell Carcinoma, *RECA-EU* Renal Cell Cancer-European Union, *TCGA-KIRC* The Cancer Genome Atlas-Kidney Renal Clear Cell Carcinoma.

**Figure 2:**
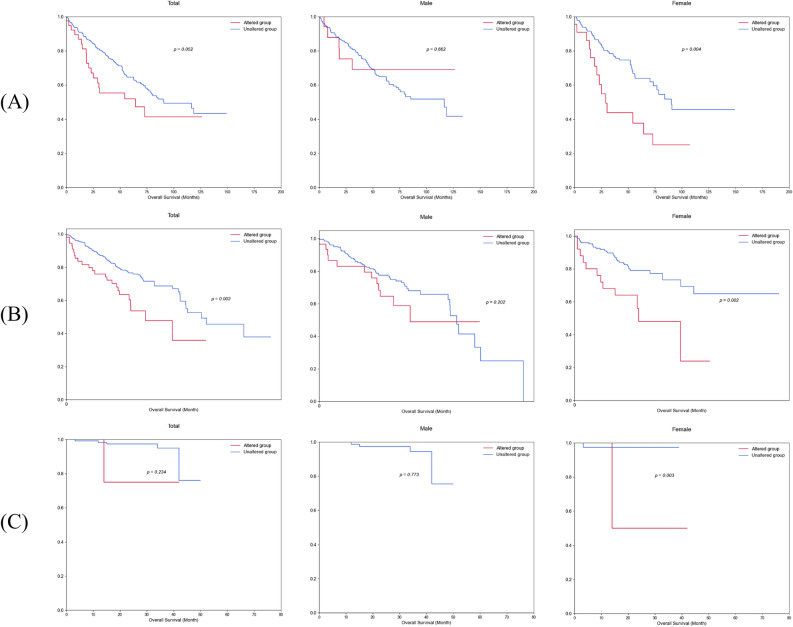
Sex differences in overall survival rates by *BAP1* mutation in the TCGA-KIRC, RECA-EU and Korean-KIRC databases. Graphs of overall survival in (**A**) TCGA-KIRC, (**B**) RECA-EU and (**C**) Korean-KIRC databases according to *BAP1* mutations were compared separately males and females. The p-values are from the Log-rank test.

**Figure 3:**
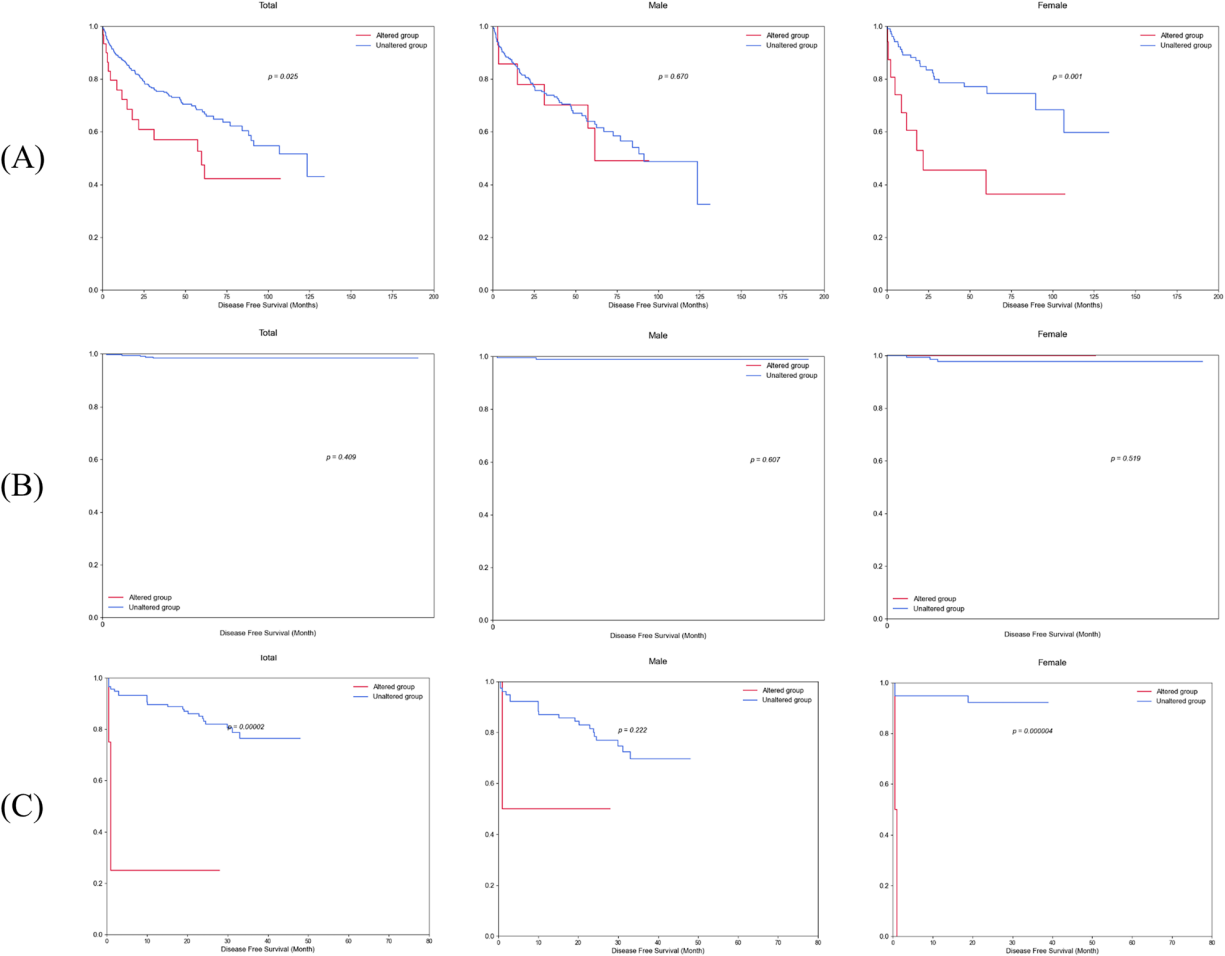
Sex differences in disease-free survival rates by *BAP1* mutation in the TCGA-KIRC, RECA-EU and Korean-KIRC databases. Graphs of disease-free survival in (**A**) TCGA-KIRC, (**B**) RECA-EU and (**C**) Korean-KIRC databases according to *BAP1* mutations were compared separately males and females. The p-values are from the Log-rank test.

Among these nine sex-dependent survival-specific genes, *ALG13, JADE3, KDM5C, KDM6A,* and *ZNF449* were X-linked genes. *ASXL3* and *KDM5C* were identified as male-specific survival genes. *ASXL3* showed male-specificity in TCGA-KIRC (OS, p = 0.017) and Korean-KIRC (OS, p = 0.005), and *KDM5C* also male-specific in RECA-EU (OS, p = 0.009) and Korean-KIRC (DFS, p = 0.016). *BAP1* and *NCOR1P1* were detected as female-specific survival genes. *BAP1* was female-specific in all three databases: TCGA-KIRC (OS, p = 0.004 and DFS, p = 0.001), RECA-EU (OS, p = 0.002) and Korean-KIRC (OS, p = 0.003 and DFS, p = 0.000004). *NCOR1P1* was also female-specific in TCGA-KIRC (DFS, p = 0.046) and RECA-EU (DFS, p = 0.00003). Figures 2 and 3 showed a comparison of the differences in survival rates by *BAP1* mutations between males and females in the three databases above.

Only female patients with a *BAP1* mutation had a lower survival rate than those without the mutation. The remaining genes (*ACSS3, ALG13, JADE3, KDM6A,* and *ZNF449*) exhibited varying patterns of sex-specific survival across the three databases. That is, *ACSS3* exhibited survival specificity in females (OS, p = 0.0001; DFS, p = 0.002) in TCGA-KIRC, but in males (DFS, p = 0.026) in Korean-KIRC. For *ALG13*, it showed female-specific survival (OS, p = 0.018) in TCGA-KIRC, while it was male-specific for OS (p = 0.010) and female-specific for DFS (p = 0.0001) in RECA-EU. *JADE3* was female-dependent in TCGA-KIRC (OS, p = 0.046), but male-dependent in RECA-EU (OS, p = 3.93E−54) and Korean-KIRC (OS, p = 0.00005 and DFS, p = 0.00002). *KDM6A* showed different sex specificity according to the databases: TCGA-KIRC (OS in female, p = 0.032), RECA-EU (OS in male, p = 0.042; DFS in female, p = 0.012), and Korean-KIRC (DFS in male, p = 0.049). Lastly, *ZNF449* showed survival specificity in females (OS, p = 0.012) in TCGA-KIRC, but in males (OS, p = 0.001) in RECA-EU. Through GSEA, the results of biological pathways and gene ontology of the sex-specific genes can be found in Supplementary Fig. S6 and Supplementary Table S14.

## Discussion

Although sex differences have been reported regardless of race or region in various carcinomas including ccRCC^1,7,8^, biological sex has not yet been evaluated as an important clinical factor in cancer treatment. Recently, with the introduction of gender medicine into oncology^5,16^, researchers’ interest in identifying sex-related genetic variations and using them as therapeutic biomarkers is increasing^5,6,9,30^.

We identified 23 sex-specific genes by comparing and analyzing 68 sex-related genes which were selected from TCGA-KIRC with RECA-EU and Korean-KIRC data. Significant differences in mutation frequencies by sex were observed when comparing these three databases We also analyzed survival differences that differed by sex among 23 sex-specific genes. Nine sex-dependent survival genes (*ACSS3, ALG13, ASXL3, BAP1, JADE3, KDM5C, KDM6A, NCOR1P1*, and *ZNF449*) were identified in at least two of three databases. *ASXL3* and *KDM5C* were finally found as male-specific survival genes in our study.

We found male-specific survival differences in *ASXL3* and *KDM5C* genes. *ASXL3*, Additional Sex Combs Like Transcriptional Regulator 3, is known to act as an adaptor protein, linking BRD4 to the BAP1 complex and regulating enhancer function in small cell lung cancer^31^. Tsuboyama et al. also reported that *ASXL3* is highly expressed and also essential for cell viability, and that inhibition of *BAP1* dramatically destabilized *ASXL3* in small cell lung cancer^32^. In this study, *ASXL3* was a male-specific survival gene in OS (TCGA-KIRC, p = 0.017; Korean-KIRC, p = 0.005). However, the sex specificity or survival specificity of *ASXL3* has not yet been reported in the literature.

*KDM5C,* Lysine Demethylase 5C, a gene located on the X chromosome, deviates from X-inactivation and causes higher mRNA expression in female tissues^6^. In our study, *KDM5C* was the only sex-specific gene in mutation frequency in at least two of the three databases. Ricketts et al. reported that *KDM5C* mutation was highly observed in male patients (p < 0.0001) in the TCGA-KIRC, Japanese, and Chinese cohorts and that it was sex-specific in TCGA-KIRC (p = 0.0039) and Chinese cohorts (p = 0.0104)^12^. Dunford et al. also reported that loss-of-function *KDM5C* mutations and copy number loss of *KDM5C* were higher in males (p < 0.0001)^6^. They identified *KDM5C* and *KDM6A* as the EXITS (escape from X-inactivation tumor suppressors) genes and suggested that mutations in EXITS genes could underlie the male predominance in various cancers. *KDM5C* was reported to have a high mutation rate in males in RCC^6,12^, and a high expression rate in females in melanoma^33^. When GSEA was conducted between sex-specific genes based on GO and KEGG gene sets, the findings revealed that *KDM5C* and *KDM6A* were significantly enriched in histone lysine demethylation (GO:0070076) (p = 0.000058173). Notably, a previous ccRCC study by Guo and Zhang reported the involvement of histone demethylase activity in RCC^34^.

Female-specific survival differences were also found in *BAP1* and *NCOR1P1* genes. *BAP1* and *NCORR1P1* were finally identified as female-specific survival genes in our study. The extraction of *BAP1* as the most important sex-related genes by all three feature selection methods coincided with our statistical verification and showed the validity of the study using artificial intelligence.

*BAP1*, BRCA1 Associated Protein 1, encodes a deubiquitylase related to multiprotein complexes that regulate cellular pathways including the cell cycle, cell differentiation, apoptosis, gluconeogenesis, and DNA damage response^35^. The *BAP1* protein acts as a tumor suppressor and is often inactivated in ccRCC^36,37^. Sex difference in *BAP1* with higher mutation frequency in females was observed in TCGA-KIRC (male:female = 5.80:14.56, p = 0.003), but not in RECA-EU (male:female = 12.24:14.69, p = 0.471) and Korean-KIRC (male:female = 2.53:4.88, p = 0.605) in this study. Similar to our results of Korean-KIRC, Ricketts et al. found higher *BAP1* mutation frequencies in females only in TCGA-KIRC, but not in Japanese and Chinese cohorts (p = 0.001)^12^. Luchini et al.^15^ reported in a systematic review with meta-analysis of ccRCC that *BAP1* mutations were mutated more often in females (p < 0.0001). Li et al.^5^ also reported that the incidence of *BAP1* mutations in ccRCC patients was higher in females (15%) than in males (6.1%). Additionally, in our study, *BAP1* was a female-specific survival gene in OS (TCGA-KIRC, p = 0.004; RECA-EU, p = 0.002; and Korean-KIRC, p = 0.003) and DFS (TCGA-KIRC, p = 0.001 and Korean-KIRC, p = 0.000004). Several studies have reported on the relationship between BAP1 mutation and survival in RCC. *BAP1* mutation was reported to be associated with significantly poorer survival in female patients (p = 0.0021) but not in male patients (p = 0.7659)^12^. Manley et al. showed that *BAP1* mutation in ccRCC was associated with decreased cancer-specific survival (p = 0.004) in a multivariable model^38^. Luchini et al.^15^ also reported that *BAP1* mutated clear cell renal carcinomas were frequently observed in females and were associated with high tumor grade (p < 0.0001), increased all-cause mortality, cancer-specific mortality, and risk of recurrence.

*NCOR1P1*, Nuclear Receptor Corepressor 1 Pseudogene 1, is predicted to enable transcription corepressor activity, and to be involved in the negative regulation of transcription by RNA polymerase II^39^. *NCOR1* has been reported as one of the nuclear receptor co-regulators, with mutations observed in hormone-dependent cancers such as breast, ovarian, and prostate cancers^40^. *NCOR1P1* was found to be a female-specific survival gene in DFS (TCGA-KIRC, p = 0.046; RECA-EU, p = 0.00003) in this study. However, there are no previous reports of sex specificity or survival specificity for *NCOR1P1* in ccRCC or other cancers.

Among the nine sex-dependent survival genes, the remaining five genes (*ACSS3, ALG13, JADE3, KDM6A* and *ZNF449*) showed different sex specificities depending on the databases, and lacked consistency. Some explanation is required as to whether this is due to chance or other factors. However, the limited information available does not sufficiently explain these contradictory results.

*ACSS3*, Acyl-CoA Synthetase Short Chain Family Member 3, is located in mitochondrial matrix and is predicted to be involved in ketone body biosynthetic process^39^. Limited information is available regarding the role of *ACSS3*, but it has been suggested as a prognostic biomarker in gastric cancer^41^. In our study, *ACSS3* was survival-specific in females in TCGA-KIRC (p = 0.0001 in OS; p = 0.002 in DFS), whereas survival-specific in males in Korean-KIRC (p = 0.026 in DFS), showing contradictory results. The association of *ACSS3* with sex or survival has not been reported in renal cancer, but it has been studied in other cancers. Zhou et al. reported that prostate cancer patients with lower *ACSS3* expression had significantly shorter DFS in both univariate (HR 0.563, 95% CI 0.36–0.89, p = 0.013) and multivariate analyses (HR 0.575, 95% CI 0.36–0.91, p = 0.018)^42^.

*ALG13*, ALG13 UDP-N-acetylglucosaminyltransferase subunit, is an enzyme involved in protein N-glycosylation, and is associated with an X-linked congenital glycosylation disorder with severe developmental delay, epilepsy and intellectual disability. In this study, *ALG13* was survival-specific in females (OS, p = 0.018) in TCGA-KIRC, whereas it was survival specific in males (OS, p = 0.010) and females (DFS, p = 0.0001) in RECA-EU. There has been no report in RCC regarding the association of *ALG13* with sex or survival specificity. However, it has been reported that patients with high *ALG13* expression had longer OS than those with low expression in non-small-cell lung cancer^43^, and *ALG13* mutations in uterine corpus endometrial carcinoma were linked with better survival (p = 0.01)^44^.

*JADE3*, Jade family PHD finger 3, participates in promoting histone acetylation during the process of transcription^45^. *JADE3* was female-dependent in TCGA-KIRC (OS, p = 0.046), whereas it was male-dependent in RECA-EU (OS, p = 3.93E−54) and in Korean-KIRC (OS, p = 0.00005 and DFS, p = 0.00002). *JADE3* has not been reported in terms of sex or survival in RCC. However, *JADE3* has been reported to be upregulated in colorectal cancer and highly associated with cancer progression, and patients with high *JADE3* expression had a shorter 5-year OS (p = 0.005)^45^.

*KDM6A*, Lysine Demethylase 6A, is located on the X chromosome and encodes the histone lysine demethylase UTX. Although *KDM6A* was survival specific in all three databases, it showed inconsistent results across the three databases in this study. It was survival-specific in females in TCGA-KIRC (p = 0.032 in OS) and RECA-EU (p = 0.012 in DFS), whereas it was male-specific in RECA-EU (p = 0.042 in OS) and Korean-KIRC (p = 0.049 in DFS). *KDM6A* has been reported as a prototypical sex-biasing tumor suppressor gene by Kaneko et al.^46^. They reported that loss of *KDM6A* consistently increased bladder cancer risk only in female knockout mice but not in male knockout mice.

*ZNF449*, Zinc Finger Protein 449, encodes a nuclear protein that likely functions as a transcription factor. Zinc Finger proteins are known to play an important role in various cell functions such as cell proliferation, differentiation, and apoptosis^47^. In our study, *ZNF449* was survival-specific in female (OS, p = 0.012) in TCGA-KIRC, but was in male (OS, p = 0.001) in RECA-EU. There are no reports of sex or survival specificity of *ZNF449* in RCC or other cancers.

Various factors have been suggested as causes of the sex disparity, including environmental factors, immunological^7^, hormonal^2,7^, genetic, and pharmacokinetic^16^ differences between males and females, X chromosome effects^1,7^, and differences in the efficiency of the immunological and genomic surveillance mechanisms between males and females^7^. Sex hormones, particularly estrogen, may play an important role in regulating cellular aging and deterioration and protecting women from some cancers^2^. At the chromosomal level, it has been suggested that X-linked mutations are more likely to have detrimental effects in male cells than in female cells, where the potential for selective inactivation of the X chromosome accompanying the mutation is favorable^2^. In particular, *KDM5C* located on the X chromosome has been reported as an X-inactivation tumor suppressor gene^6^, which may explain parts of the sex difference. However, further study is needed to find out the precise mechanism of the genes that show sex differences in patients with ccRCC.

Although we performed the analysis using various genomic databases of American, European, and Korean patients with kidney cancer, this study has several limitations. First, genetic differences arising from racial factors may have contributed to the inconsistent sex-specificity between the three databases. Second, the difference in cancer subtypes included in the three databases selected for this study may have an effect. Only ccRCC data were included in TCGA-KIRC and Korean-KIRC, whereas various renal cancers, including ccRCC, were used in RECA-EU.

## Conclusion

We discovered and validated sex-specific survival genes in patients with ccRCC by performing machine learning and NGS analyses the TCGA-KIRC, RECA-EU and Korean-KIRC databases. Genetic variants showing sex-specific survival differences were identified. Female-specific survival differences were found in *BAP1* and *NCOR1P1*. Male-specific survival differences were found in *ASXL3* and *KDM5C*. These results suggest that biological sex should be considered an important predictor in ccRCC. In the era of precision medicine, it is necessary to understand sex differences and apply this knowledge to tailor personalized medicine, rather than relying on existing treatment strategies that apply to all patients regardless of sex. Sex-specific customized treatments may improve patient survival in ccRCC.

### Supplementary Information

Supplementary Information 1.

Supplementary Information 2.

Supplementary Information 3.

Supplementary Information 4.

Supplementary Figures.

Supplementary Tables.

## Data Availability

All data generated or analyzed during this study are included in this published article and its supplementary information files.
