# Supplementary material for: Sex-specific survival gene mutations are discovered as clinical predictors of clear cell renal cell carcinoma
Source: Sci Rep. 2024 Jul 9;14:15800. doi: 10.1038/s41598-024-66525-9 (PMC11233666; doi:10.1038/s41598-024-66525-9)
Supplement: Supplementary file 5 — Supplementary Figures. [file 41598_2024_66525_MOESM5_ESM.pptx]

## Slide 1
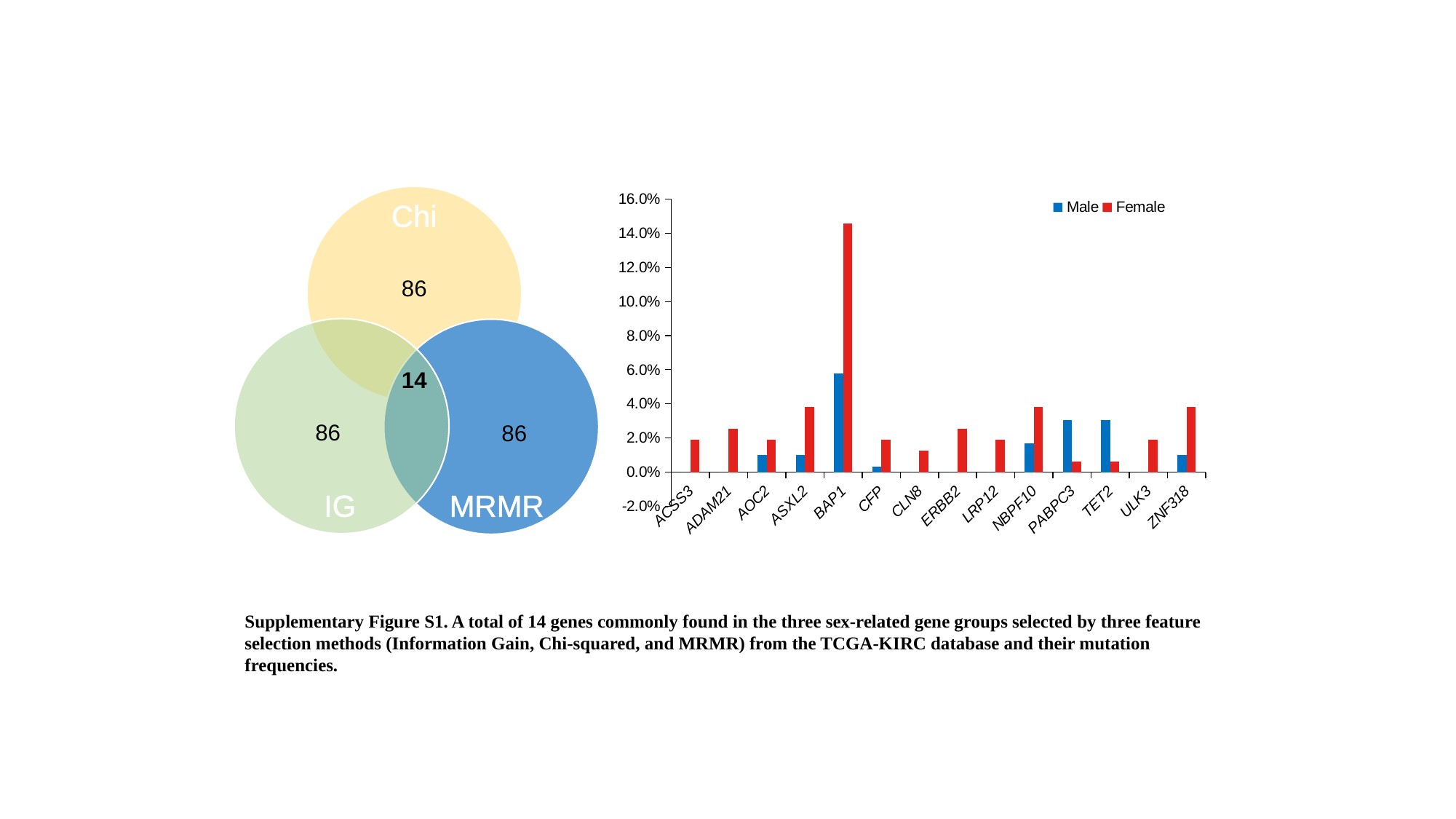

### Chart
| Category | Male | Female |
|---|---|---|
| ACSS3 | 0.0 | 0.019 |
| ADAM21 | 0.0 | 0.0253 |
| AOC2 | 0.0102 | 0.019 |
| ASXL2 | 0.0102 | 0.038 |
| BAP1 | 0.058 | 0.1456 |
| CFP | 0.0034 | 0.019 |
| CLN8 | 0.0 | 0.0127 |
| ERBB2 | 0.0 | 0.0253 |
| LRP12 | 0.0 | 0.019 |
| NBPF10 | 0.0171 | 0.038 |
| PABPC3 | 0.0307 | 0.0063 |
| TET2 | 0.0307 | 0.0063 |
| ULK3 | 0.0 | 0.019 |
| ZNF318 | 0.0102 | 0.038 |
86
Chi
 86
86
14
IG
MRMR
Supplementary Figure S1. A total of 14 genes commonly found in the three sex-related gene groups selected by three feature selection methods (Information Gain, Chi-squared, and MRMR) from the TCGA-KIRC database and their mutation frequencies.

## Slide 2
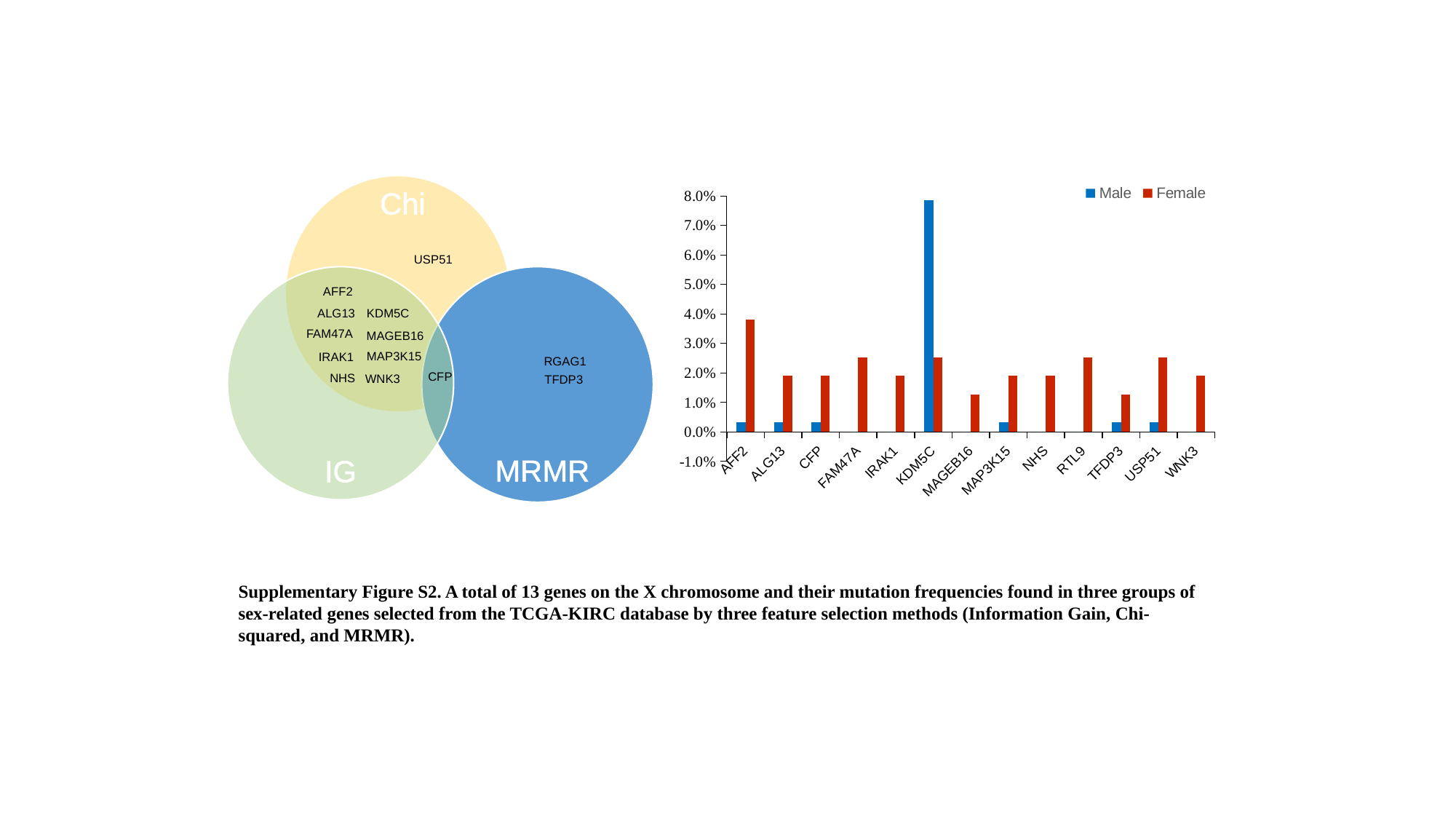

### Chart
| Category | Male | Female |
|---|---|---|
| AFF2 | 0.0034 | 0.038 |
| ALG13 | 0.0034 | 0.019 |
| CFP | 0.0034 | 0.019 |
| FAM47A | 0.0 | 0.0253 |
| IRAK1 | 0.0 | 0.019 |
| KDM5C | 0.0785 | 0.0253 |
| MAGEB16 | 0.0 | 0.0127 |
| MAP3K15 | 0.0034 | 0.019 |
| NHS | 0.0 | 0.019 |
| RTL9 | 0.0 | 0.0253 |
| TFDP3 | 0.0034 | 0.0127 |
| USP51 | 0.0034 | 0.0253 |
| WNK3 | 0.0 | 0.019 |
Chi
USP51
AFF2
ALG13
KDM5C
FAM47A
MAGEB16
MAP3K15
IRAK1
RGAG1
CFP
NHS
WNK3
TFDP3
MRMR
IG
Supplementary Figure S2. A total of 13 genes on the X chromosome and their mutation frequencies found in three groups of sex-related genes selected from the TCGA-KIRC database by three feature selection methods (Information Gain, Chi-squared, and MRMR).

## Slide 3
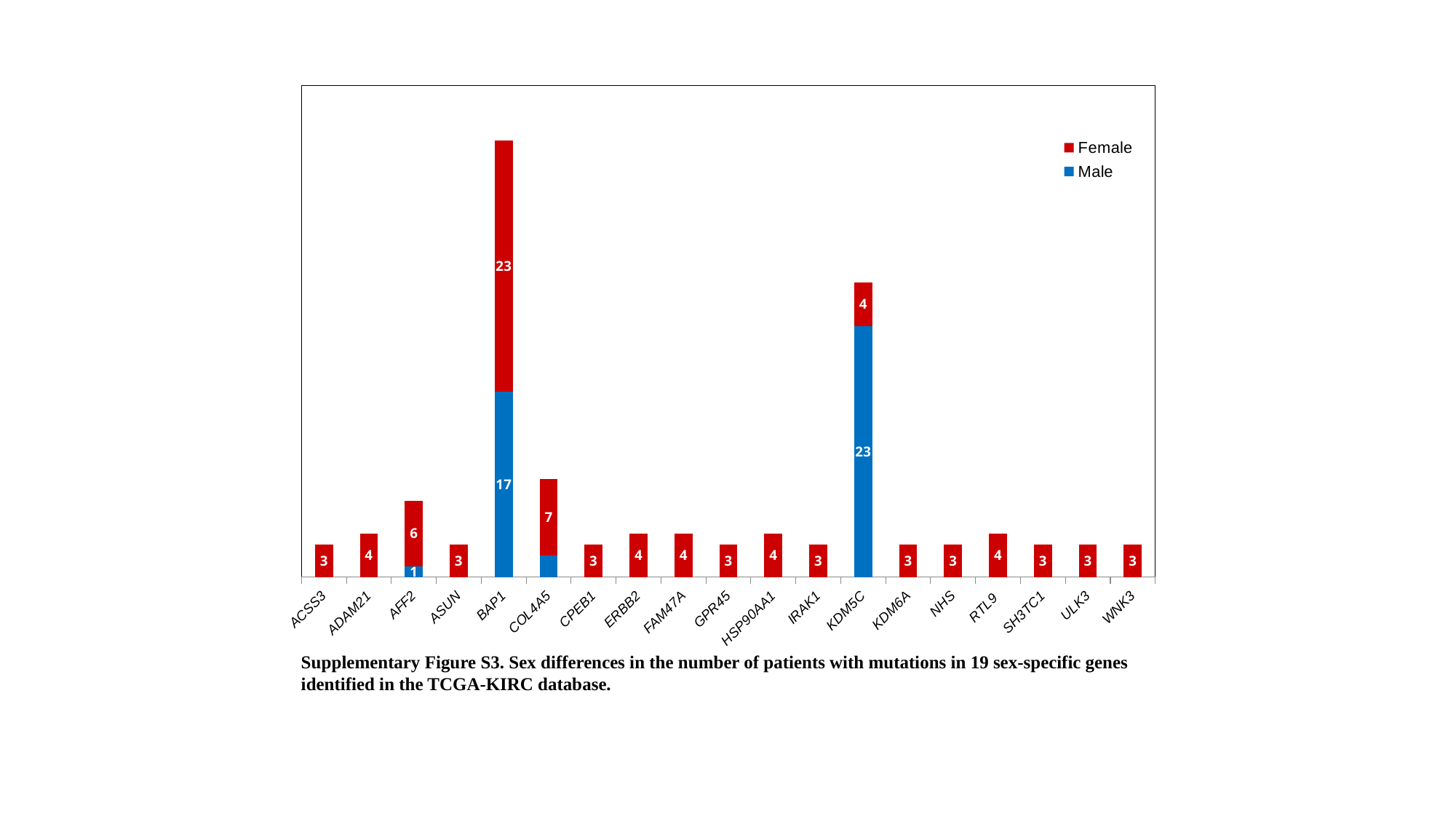

### Chart
| Category | Male | Female |
|---|---|---|
| ACSS3 | None | 3.0 |
| ADAM21 | None | 4.0 |
| AFF2 | 1.0 | 6.0 |
| ASUN | None | 3.0 |
| BAP1 | 17.0 | 23.0 |
| COL4A5 | 2.0 | 7.0 |
| CPEB1 | None | 3.0 |
| ERBB2 | None | 4.0 |
| FAM47A | None | 4.0 |
| GPR45 | None | 3.0 |
| HSP90AA1 | None | 4.0 |
| IRAK1 | None | 3.0 |
| KDM5C | 23.0 | 4.0 |
| KDM6A | None | 3.0 |
| NHS | None | 3.0 |
| RTL9 | None | 4.0 |
| SH3TC1 | None | 3.0 |
| ULK3 | None | 3.0 |
| WNK3 | None | 3.0 |Supplementary Figure S3. Sex differences in the number of patients with mutations in 19 sex-specific genes identified in the TCGA-KIRC database.

## Slide 4
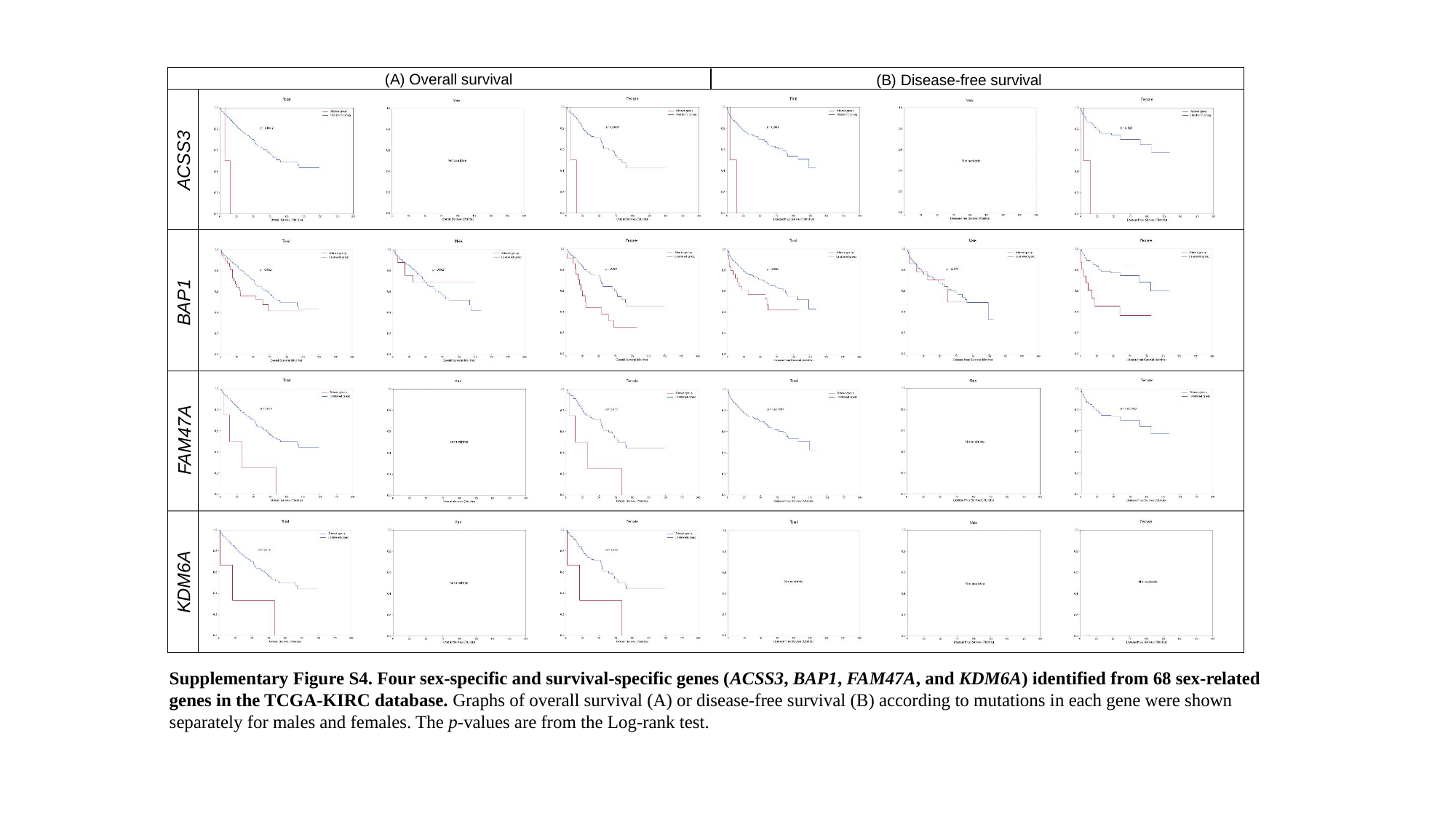

(A) Overall survival
(B) Disease-free survival
ACSS3
BAP1
FAM47A
KDM6A
Supplementary Figure S4. Four sex-specific and survival-specific genes (ACSS3, BAP1, FAM47A, and KDM6A) identified from 68 sex-related genes in the TCGA-KIRC database. Graphs of overall survival (A) or disease-free survival (B) according to mutations in each gene were shown separately for males and females. The p-values are from the Log-rank test.

## Slide 5
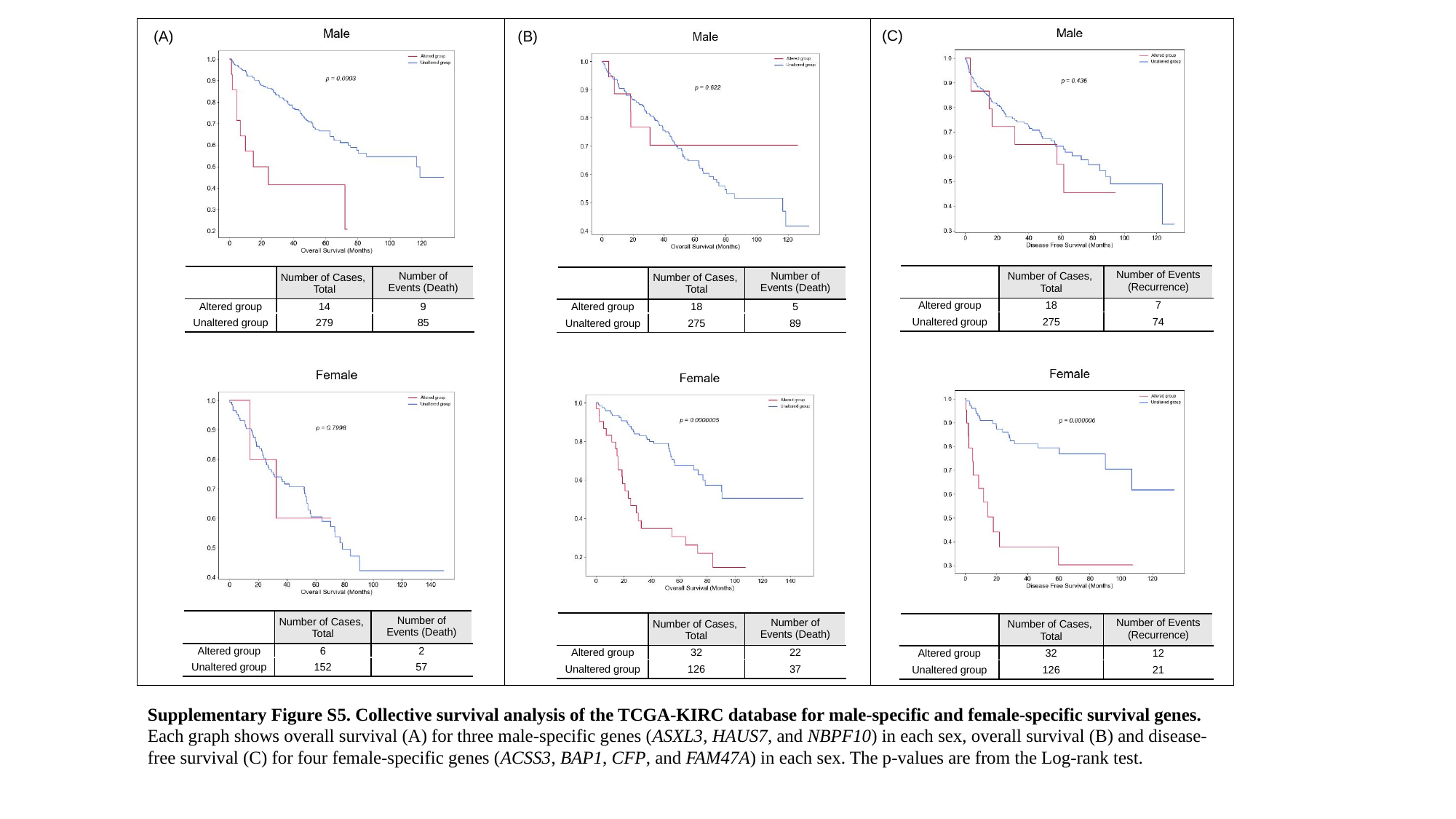

(C)
(A)
(B)
| | Number of Cases, Total | Number of Events (Recurrence) |
| --- | --- | --- |
| Altered group | 18 | 7 |
| Unaltered group | 275 | 74 |
| | Number of Cases, Total | Number of Events (Death) |
| --- | --- | --- |
| Altered group | 14 | 9 |
| Unaltered group | 279 | 85 |
| | Number of Cases, Total | Number of Events (Death) |
| --- | --- | --- |
| Altered group | 18 | 5 |
| Unaltered group | 275 | 89 |
| | Number of Cases, Total | Number of Events (Death) |
| --- | --- | --- |
| Altered group | 6 | 2 |
| Unaltered group | 152 | 57 |
| | Number of Cases, Total | Number of Events (Death) |
| --- | --- | --- |
| Altered group | 32 | 22 |
| Unaltered group | 126 | 37 |
| | Number of Cases, Total | Number of Events (Recurrence) |
| --- | --- | --- |
| Altered group | 32 | 12 |
| Unaltered group | 126 | 21 |
Supplementary Figure S5. Collective survival analysis of the TCGA-KIRC database for male-specific and female-specific survival genes. Each graph shows overall survival (A) for three male-specific genes (ASXL3, HAUS7, and NBPF10) in each sex, overall survival (B) and disease-free survival (C) for four female-specific genes (ACSS3, BAP1, CFP, and FAM47A) in each sex. The p-values are from the Log-rank test.

## Slide 6
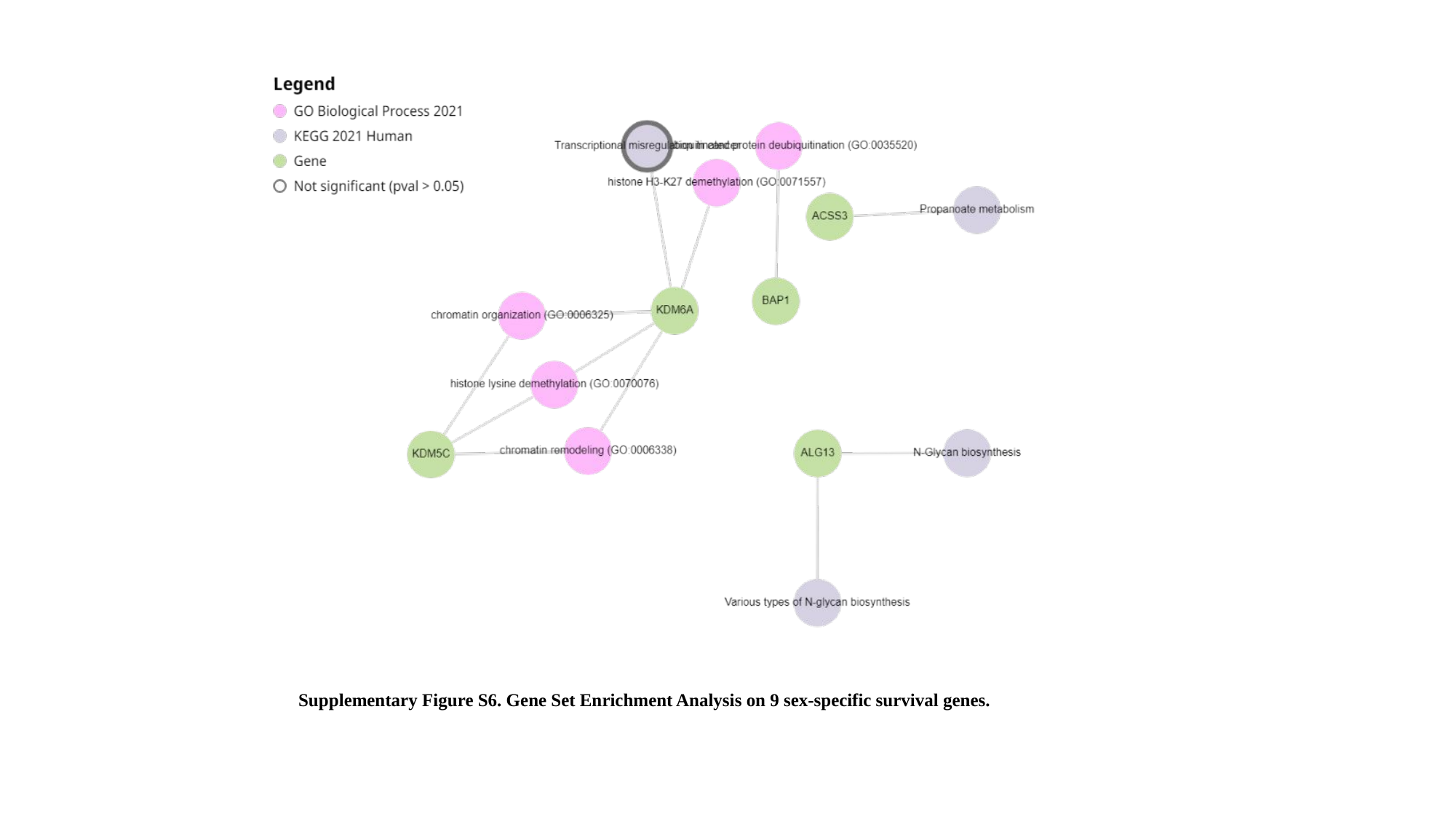

Supplementary Figure S6. Gene Set Enrichment Analysis on 9 sex-specific survival genes.
