# Supplementary material for: Sex-specific survival gene mutations are discovered as clinical predictors of clear cell renal cell carcinoma
Source: Sci Rep. 2024 Jul 9;14:15800. doi: 10.1038/s41598-024-66525-9 (PMC11233666; doi:10.1038/s41598-024-66525-9)
Supplement: Supplementary file 6 — Supplementary Tables. [file 41598_2024_66525_MOESM6_ESM.pptx]

## Slide 1
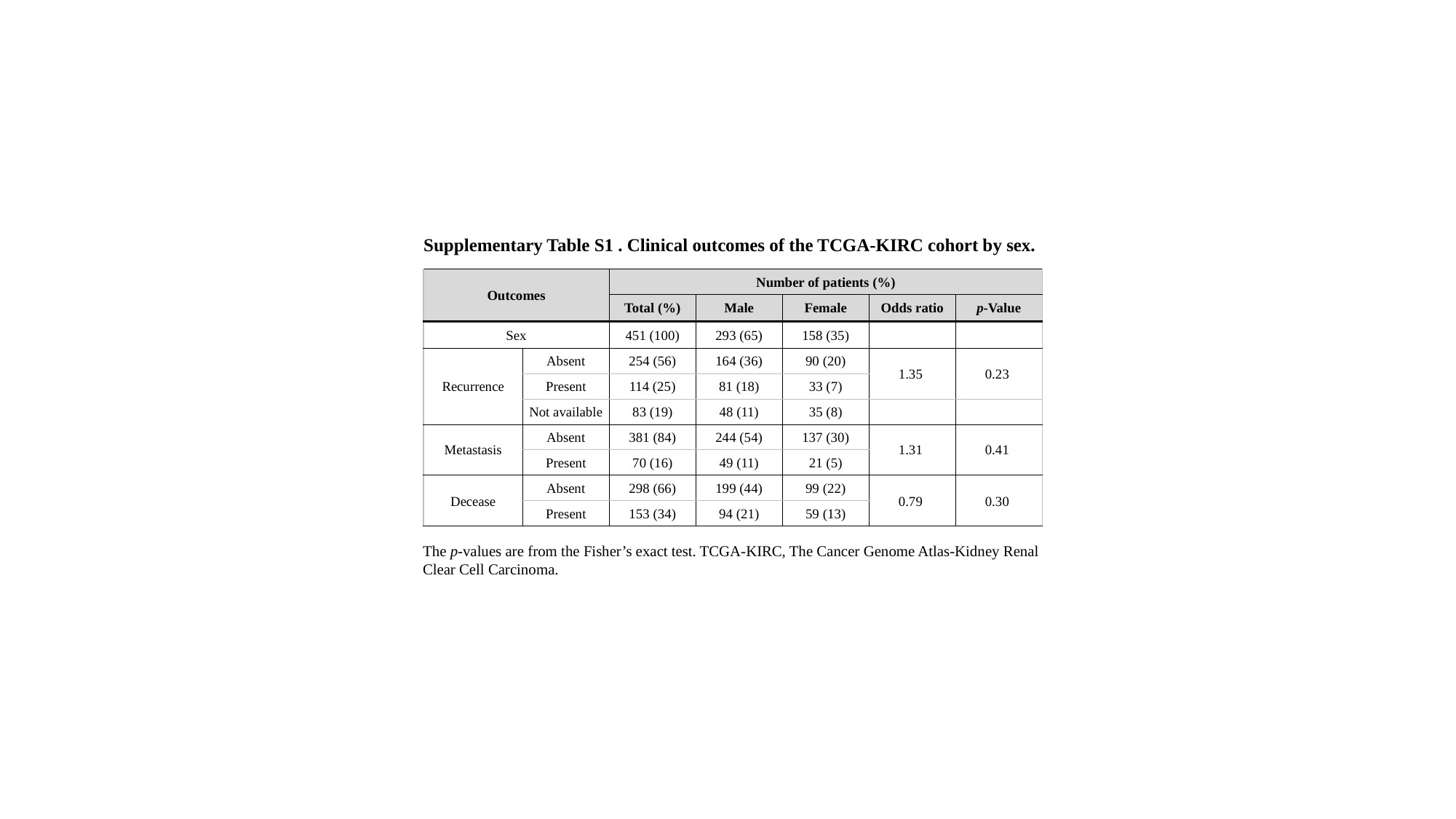

Supplementary Table S1 . Clinical outcomes of the TCGA-KIRC cohort by sex.
| Outcomes | | Number of patients (%) | | | | |
| --- | --- | --- | --- | --- | --- | --- |
| | | Total (%) | Male | Female | Odds ratio | p-Value |
| Sex | | 451 (100) | 293 (65) | 158 (35) | | |
| Recurrence | Absent | 254 (56) | 164 (36) | 90 (20) | 1.35 | 0.23 |
| | Present | 114 (25) | 81 (18) | 33 (7) | | |
| | Not available | 83 (19) | 48 (11) | 35 (8) | | |
| Metastasis | Absent | 381 (84) | 244 (54) | 137 (30) | 1.31 | 0.41 |
| | Present | 70 (16) | 49 (11) | 21 (5) | | |
| Decease | Absent | 298 (66) | 199 (44) | 99 (22) | 0.79 | 0.30 |
| | Present | 153 (34) | 94 (21) | 59 (13) | | |
The p-values are from the Fisher’s exact test. TCGA-KIRC, The Cancer Genome Atlas-Kidney Renal Clear Cell Carcinoma.

## Slide 2
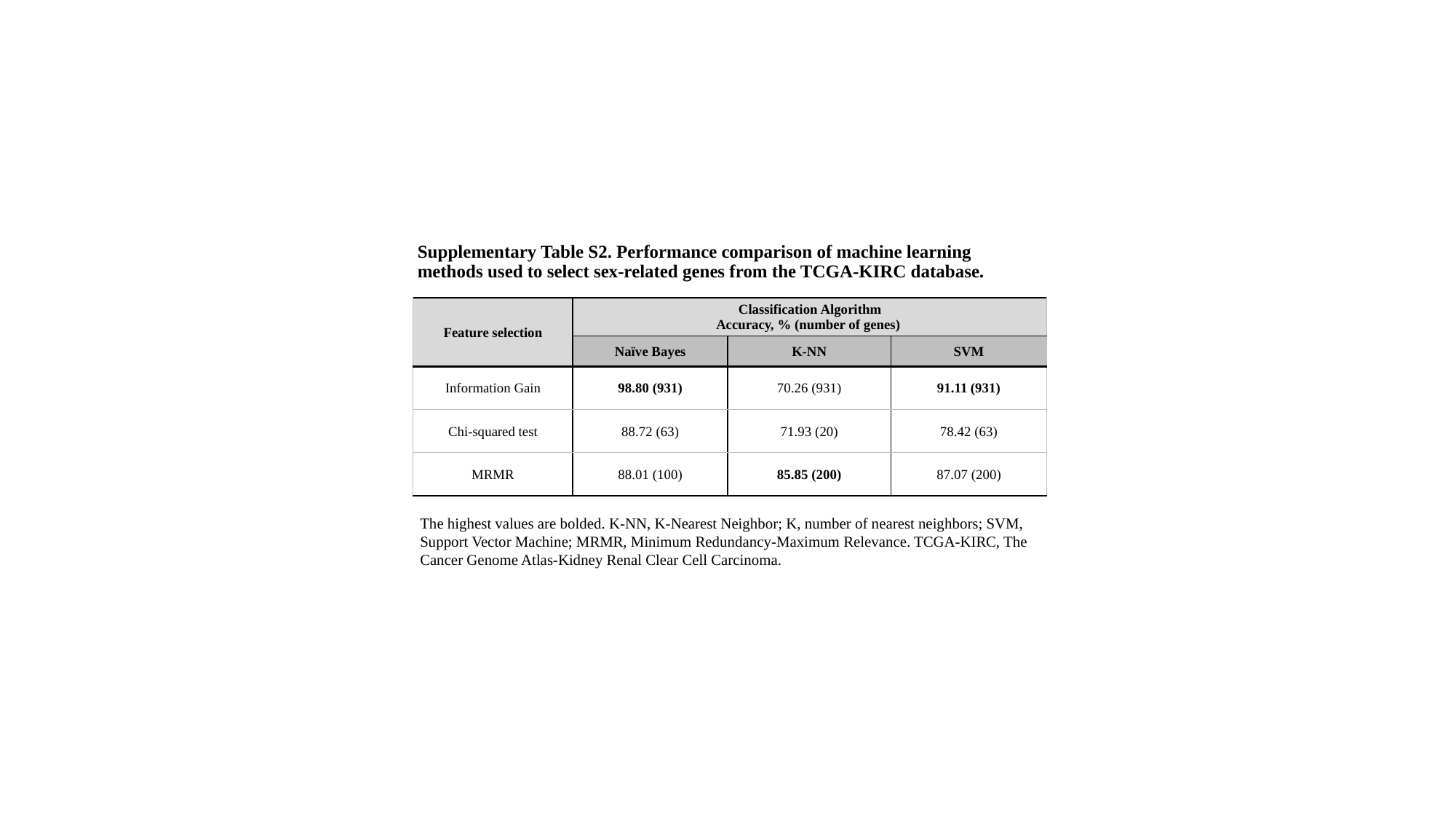

Supplementary Table S2. Performance comparison of machine learning methods used to select sex-related genes from the TCGA-KIRC database.
| Feature selection | Classification Algorithm Accuracy, % (number of genes) | | |
| --- | --- | --- | --- |
| | Naïve Bayes | K-NN | SVM |
| Information Gain | 98.80 (931) | 70.26 (931) | 91.11 (931) |
| Chi-squared test | 88.72 (63) | 71.93 (20) | 78.42 (63) |
| MRMR | 88.01 (100) | 85.85 (200) | 87.07 (200) |
The highest values are bolded. K-NN, K-Nearest Neighbor; K, number of nearest neighbors; SVM, Support Vector Machine; MRMR, Minimum Redundancy-Maximum Relevance. TCGA-KIRC, The Cancer Genome Atlas-Kidney Renal Clear Cell Carcinoma.

## Slide 3
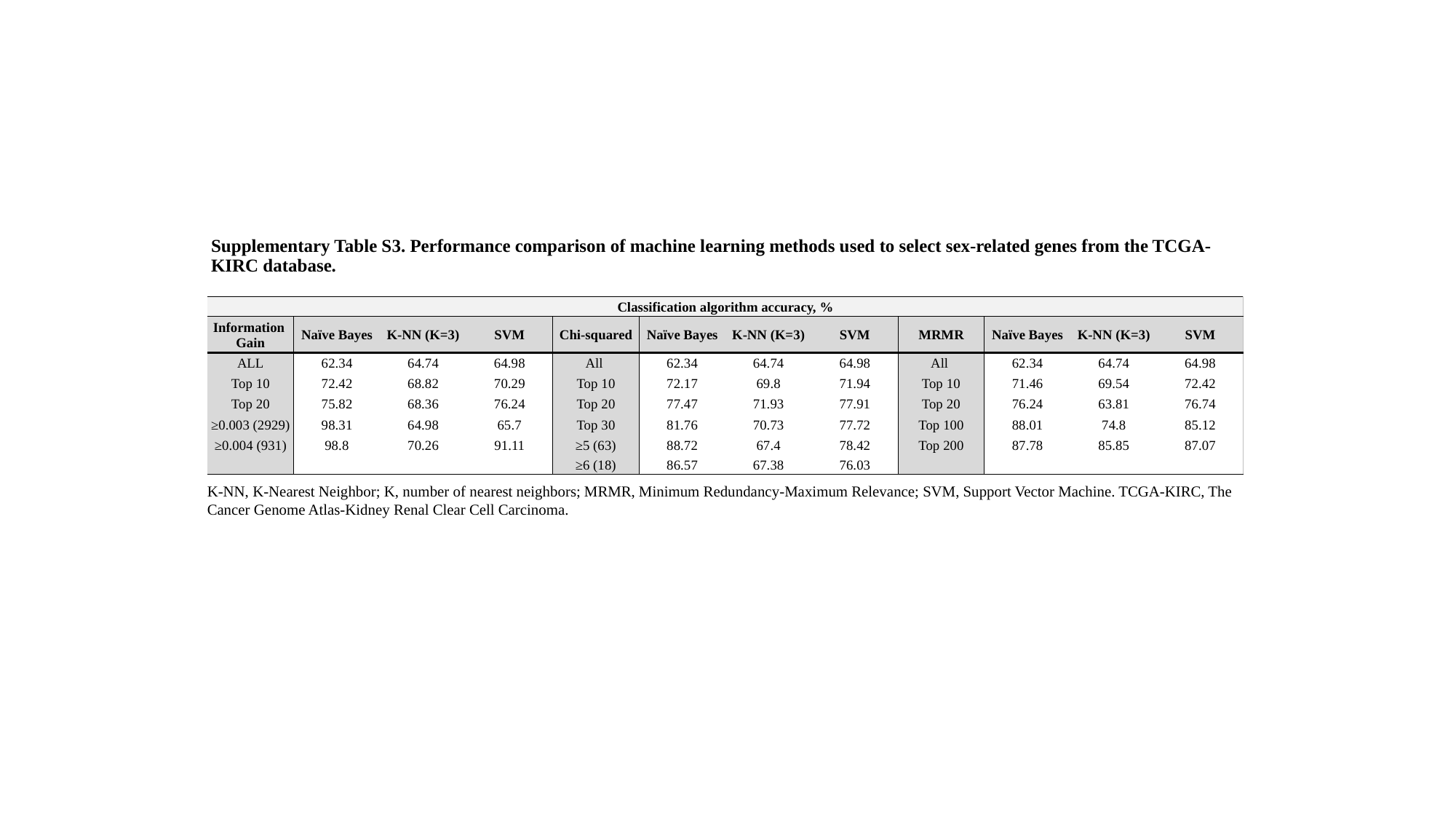

Supplementary Table S3. Performance comparison of machine learning methods used to select sex-related genes from the TCGA-KIRC database.
| Classification algorithm accuracy, % | | | | | | | | | | | |
| --- | --- | --- | --- | --- | --- | --- | --- | --- | --- | --- | --- |
| Information Gain | Naïve Bayes | K-NN (K=3) | SVM | Chi-squared | Naïve Bayes | K-NN (K=3) | SVM | MRMR | Naïve Bayes | K-NN (K=3) | SVM |
| ALL | 62.34 | 64.74 | 64.98 | All | 62.34 | 64.74 | 64.98 | All | 62.34 | 64.74 | 64.98 |
| Top 10 | 72.42 | 68.82 | 70.29 | Top 10 | 72.17 | 69.8 | 71.94 | Top 10 | 71.46 | 69.54 | 72.42 |
| Top 20 | 75.82 | 68.36 | 76.24 | Top 20 | 77.47 | 71.93 | 77.91 | Top 20 | 76.24 | 63.81 | 76.74 |
| ≥0.003 (2929) | 98.31 | 64.98 | 65.7 | Top 30 | 81.76 | 70.73 | 77.72 | Top 100 | 88.01 | 74.8 | 85.12 |
| ≥0.004 (931) | 98.8 | 70.26 | 91.11 | ≥5 (63) | 88.72 | 67.4 | 78.42 | Top 200 | 87.78 | 85.85 | 87.07 |
| | | | | ≥6 (18) | 86.57 | 67.38 | 76.03 | | | | |
K-NN, K-Nearest Neighbor; K, number of nearest neighbors; MRMR, Minimum Redundancy-Maximum Relevance; SVM, Support Vector Machine. TCGA-KIRC, The Cancer Genome Atlas-Kidney Renal Clear Cell Carcinoma.

## Slide 4
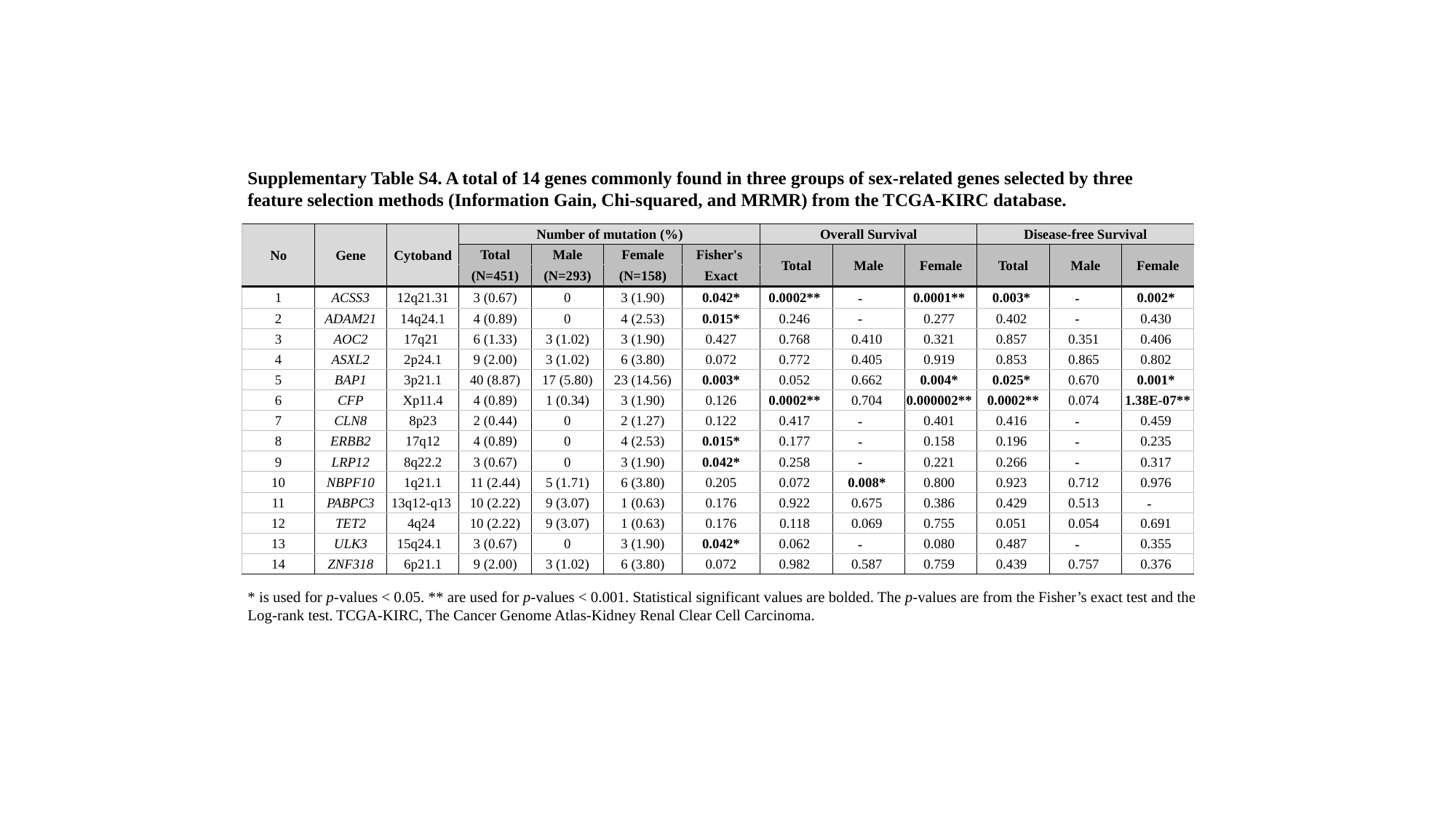

Supplementary Table S4. A total of 14 genes commonly found in three groups of sex-related genes selected by three feature selection methods (Information Gain, Chi-squared, and MRMR) from the TCGA-KIRC database.
| No | Gene | Cytoband | Number of mutation (%) | | | | Overall Survival | | | Disease-free Survival | | |
| --- | --- | --- | --- | --- | --- | --- | --- | --- | --- | --- | --- | --- |
| | | | Total | Male | Female | Fisher's | Total | Male | Female | Total | Male | Female |
| | | | (N=451) | (N=293) | (N=158) | Exact | | | | | | |
| 1 | ACSS3 | 12q21.31 | 3 (0.67) | 0 | 3 (1.90) | 0.042\* | 0.0002\*\* | - | 0.0001\*\* | 0.003\* | - | 0.002\* |
| 2 | ADAM21 | 14q24.1 | 4 (0.89) | 0 | 4 (2.53) | 0.015\* | 0.246 | - | 0.277 | 0.402 | - | 0.430 |
| 3 | AOC2 | 17q21 | 6 (1.33) | 3 (1.02) | 3 (1.90) | 0.427 | 0.768 | 0.410 | 0.321 | 0.857 | 0.351 | 0.406 |
| 4 | ASXL2 | 2p24.1 | 9 (2.00) | 3 (1.02) | 6 (3.80) | 0.072 | 0.772 | 0.405 | 0.919 | 0.853 | 0.865 | 0.802 |
| 5 | BAP1 | 3p21.1 | 40 (8.87) | 17 (5.80) | 23 (14.56) | 0.003\* | 0.052 | 0.662 | 0.004\* | 0.025\* | 0.670 | 0.001\* |
| 6 | CFP | Xp11.4 | 4 (0.89) | 1 (0.34) | 3 (1.90) | 0.126 | 0.0002\*\* | 0.704 | 0.000002\*\* | 0.0002\*\* | 0.074 | 1.38E-07\*\* |
| 7 | CLN8 | 8p23 | 2 (0.44) | 0 | 2 (1.27) | 0.122 | 0.417 | - | 0.401 | 0.416 | - | 0.459 |
| 8 | ERBB2 | 17q12 | 4 (0.89) | 0 | 4 (2.53) | 0.015\* | 0.177 | - | 0.158 | 0.196 | - | 0.235 |
| 9 | LRP12 | 8q22.2 | 3 (0.67) | 0 | 3 (1.90) | 0.042\* | 0.258 | - | 0.221 | 0.266 | - | 0.317 |
| 10 | NBPF10 | 1q21.1 | 11 (2.44) | 5 (1.71) | 6 (3.80) | 0.205 | 0.072 | 0.008\* | 0.800 | 0.923 | 0.712 | 0.976 |
| 11 | PABPC3 | 13q12-q13 | 10 (2.22) | 9 (3.07) | 1 (0.63) | 0.176 | 0.922 | 0.675 | 0.386 | 0.429 | 0.513 | - |
| 12 | TET2 | 4q24 | 10 (2.22) | 9 (3.07) | 1 (0.63) | 0.176 | 0.118 | 0.069 | 0.755 | 0.051 | 0.054 | 0.691 |
| 13 | ULK3 | 15q24.1 | 3 (0.67) | 0 | 3 (1.90) | 0.042\* | 0.062 | - | 0.080 | 0.487 | - | 0.355 |
| 14 | ZNF318 | 6p21.1 | 9 (2.00) | 3 (1.02) | 6 (3.80) | 0.072 | 0.982 | 0.587 | 0.759 | 0.439 | 0.757 | 0.376 |
* is used for p-values < 0.05. ** are used for p-values < 0.001. Statistical significant values are bolded. The p-values are from the Fisher’s exact test and the Log-rank test. TCGA-KIRC, The Cancer Genome Atlas-Kidney Renal Clear Cell Carcinoma.

## Slide 5
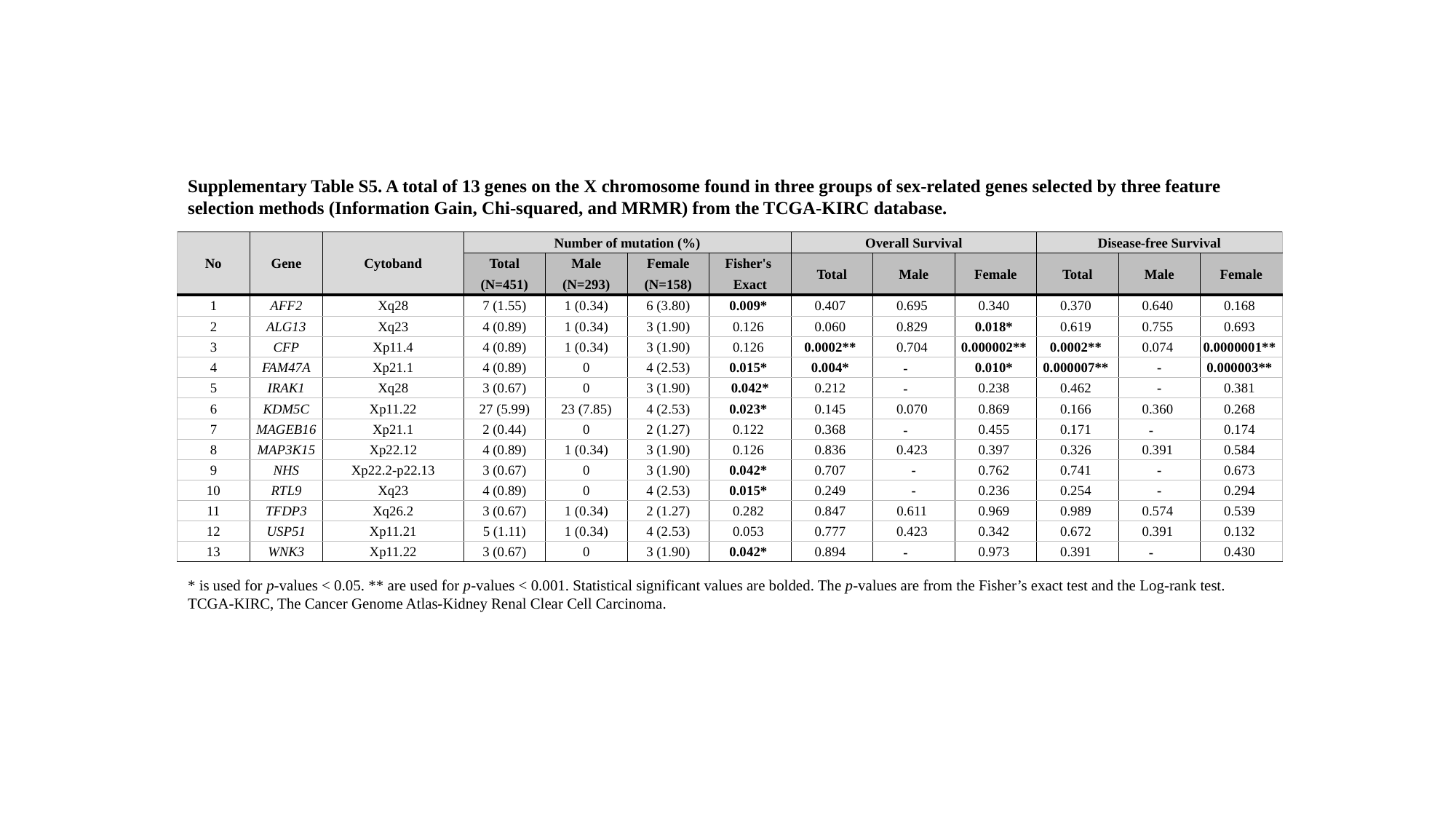

Supplementary Table S5. A total of 13 genes on the X chromosome found in three groups of sex-related genes selected by three feature selection methods (Information Gain, Chi-squared, and MRMR) from the TCGA-KIRC database.
| No | Gene | Cytoband | Number of mutation (%) | | | | Overall Survival | | | Disease-free Survival | | |
| --- | --- | --- | --- | --- | --- | --- | --- | --- | --- | --- | --- | --- |
| | | | Total | Male | Female | Fisher's | Total | Male | Female | Total | Male | Female |
| | | | (N=451) | (N=293) | (N=158) | Exact | | | | | | |
| 1 | AFF2 | Xq28 | 7 (1.55) | 1 (0.34) | 6 (3.80) | 0.009\* | 0.407 | 0.695 | 0.340 | 0.370 | 0.640 | 0.168 |
| 2 | ALG13 | Xq23 | 4 (0.89) | 1 (0.34) | 3 (1.90) | 0.126 | 0.060 | 0.829 | 0.018\* | 0.619 | 0.755 | 0.693 |
| 3 | CFP | Xp11.4 | 4 (0.89) | 1 (0.34) | 3 (1.90) | 0.126 | 0.0002\*\* | 0.704 | 0.000002\*\* | 0.0002\*\* | 0.074 | 0.0000001\*\* |
| 4 | FAM47A | Xp21.1 | 4 (0.89) | 0 | 4 (2.53) | 0.015\* | 0.004\* | - | 0.010\* | 0.000007\*\* | - | 0.000003\*\* |
| 5 | IRAK1 | Xq28 | 3 (0.67) | 0 | 3 (1.90) | 0.042\* | 0.212 | - | 0.238 | 0.462 | - | 0.381 |
| 6 | KDM5C | Xp11.22 | 27 (5.99) | 23 (7.85) | 4 (2.53) | 0.023\* | 0.145 | 0.070 | 0.869 | 0.166 | 0.360 | 0.268 |
| 7 | MAGEB16 | Xp21.1 | 2 (0.44) | 0 | 2 (1.27) | 0.122 | 0.368 | - | 0.455 | 0.171 | - | 0.174 |
| 8 | MAP3K15 | Xp22.12 | 4 (0.89) | 1 (0.34) | 3 (1.90) | 0.126 | 0.836 | 0.423 | 0.397 | 0.326 | 0.391 | 0.584 |
| 9 | NHS | Xp22.2-p22.13 | 3 (0.67) | 0 | 3 (1.90) | 0.042\* | 0.707 | - | 0.762 | 0.741 | - | 0.673 |
| 10 | RTL9 | Xq23 | 4 (0.89) | 0 | 4 (2.53) | 0.015\* | 0.249 | - | 0.236 | 0.254 | - | 0.294 |
| 11 | TFDP3 | Xq26.2 | 3 (0.67) | 1 (0.34) | 2 (1.27) | 0.282 | 0.847 | 0.611 | 0.969 | 0.989 | 0.574 | 0.539 |
| 12 | USP51 | Xp11.21 | 5 (1.11) | 1 (0.34) | 4 (2.53) | 0.053 | 0.777 | 0.423 | 0.342 | 0.672 | 0.391 | 0.132 |
| 13 | WNK3 | Xp11.22 | 3 (0.67) | 0 | 3 (1.90) | 0.042\* | 0.894 | - | 0.973 | 0.391 | - | 0.430 |
* is used for p-values < 0.05. ** are used for p-values < 0.001. Statistical significant values are bolded. The p-values are from the Fisher’s exact test and the Log-rank test. TCGA-KIRC, The Cancer Genome Atlas-Kidney Renal Clear Cell Carcinoma.

## Slide 6
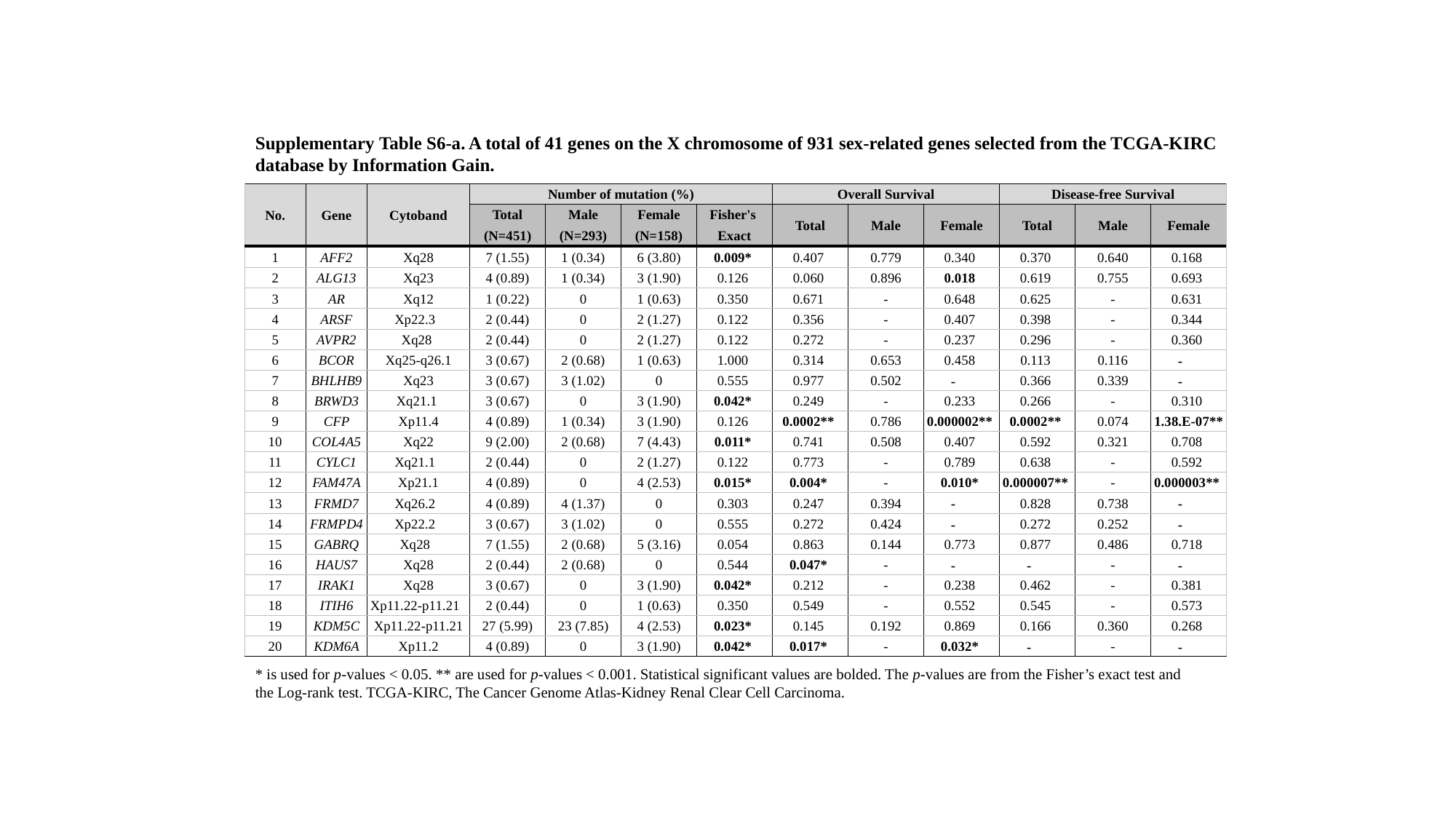

Supplementary Table S6-a. A total of 41 genes on the X chromosome of 931 sex-related genes selected from the TCGA-KIRC database by Information Gain.
| No. | Gene | Cytoband | Number of mutation (%) | | | | Overall Survival | | | Disease-free Survival | | |
| --- | --- | --- | --- | --- | --- | --- | --- | --- | --- | --- | --- | --- |
| | | | Total | Male | Female | Fisher's | Total | Male | Female | Total | Male | Female |
| | | | (N=451) | (N=293) | (N=158) | Exact | | | | | | |
| 1 | AFF2 | Xq28 | 7 (1.55) | 1 (0.34) | 6 (3.80) | 0.009\* | 0.407 | 0.779 | 0.340 | 0.370 | 0.640 | 0.168 |
| 2 | ALG13 | Xq23 | 4 (0.89) | 1 (0.34) | 3 (1.90) | 0.126 | 0.060 | 0.896 | 0.018 | 0.619 | 0.755 | 0.693 |
| 3 | AR | Xq12 | 1 (0.22) | 0 | 1 (0.63) | 0.350 | 0.671 | - | 0.648 | 0.625 | - | 0.631 |
| 4 | ARSF | Xp22.3 | 2 (0.44) | 0 | 2 (1.27) | 0.122 | 0.356 | - | 0.407 | 0.398 | - | 0.344 |
| 5 | AVPR2 | Xq28 | 2 (0.44) | 0 | 2 (1.27) | 0.122 | 0.272 | - | 0.237 | 0.296 | - | 0.360 |
| 6 | BCOR | Xq25-q26.1 | 3 (0.67) | 2 (0.68) | 1 (0.63) | 1.000 | 0.314 | 0.653 | 0.458 | 0.113 | 0.116 | - |
| 7 | BHLHB9 | Xq23 | 3 (0.67) | 3 (1.02) | 0 | 0.555 | 0.977 | 0.502 | - | 0.366 | 0.339 | - |
| 8 | BRWD3 | Xq21.1 | 3 (0.67) | 0 | 3 (1.90) | 0.042\* | 0.249 | - | 0.233 | 0.266 | - | 0.310 |
| 9 | CFP | Xp11.4 | 4 (0.89) | 1 (0.34) | 3 (1.90) | 0.126 | 0.0002\*\* | 0.786 | 0.000002\*\* | 0.0002\*\* | 0.074 | 1.38.E-07\*\* |
| 10 | COL4A5 | Xq22 | 9 (2.00) | 2 (0.68) | 7 (4.43) | 0.011\* | 0.741 | 0.508 | 0.407 | 0.592 | 0.321 | 0.708 |
| 11 | CYLC1 | Xq21.1 | 2 (0.44) | 0 | 2 (1.27) | 0.122 | 0.773 | - | 0.789 | 0.638 | - | 0.592 |
| 12 | FAM47A | Xp21.1 | 4 (0.89) | 0 | 4 (2.53) | 0.015\* | 0.004\* | - | 0.010\* | 0.000007\*\* | - | 0.000003\*\* |
| 13 | FRMD7 | Xq26.2 | 4 (0.89) | 4 (1.37) | 0 | 0.303 | 0.247 | 0.394 | - | 0.828 | 0.738 | - |
| 14 | FRMPD4 | Xp22.2 | 3 (0.67) | 3 (1.02) | 0 | 0.555 | 0.272 | 0.424 | - | 0.272 | 0.252 | - |
| 15 | GABRQ | Xq28 | 7 (1.55) | 2 (0.68) | 5 (3.16) | 0.054 | 0.863 | 0.144 | 0.773 | 0.877 | 0.486 | 0.718 |
| 16 | HAUS7 | Xq28 | 2 (0.44) | 2 (0.68) | 0 | 0.544 | 0.047\* | - | - | - | - | - |
| 17 | IRAK1 | Xq28 | 3 (0.67) | 0 | 3 (1.90) | 0.042\* | 0.212 | - | 0.238 | 0.462 | - | 0.381 |
| 18 | ITIH6 | Xp11.22-p11.21 | 2 (0.44) | 0 | 1 (0.63) | 0.350 | 0.549 | - | 0.552 | 0.545 | - | 0.573 |
| 19 | KDM5C | Xp11.22-p11.21 | 27 (5.99) | 23 (7.85) | 4 (2.53) | 0.023\* | 0.145 | 0.192 | 0.869 | 0.166 | 0.360 | 0.268 |
| 20 | KDM6A | Xp11.2 | 4 (0.89) | 0 | 3 (1.90) | 0.042\* | 0.017\* | - | 0.032\* | - | - | - |
* is used for p-values < 0.05. ** are used for p-values < 0.001. Statistical significant values are bolded. The p-values are from the Fisher’s exact test and the Log-rank test. TCGA-KIRC, The Cancer Genome Atlas-Kidney Renal Clear Cell Carcinoma.

## Slide 7
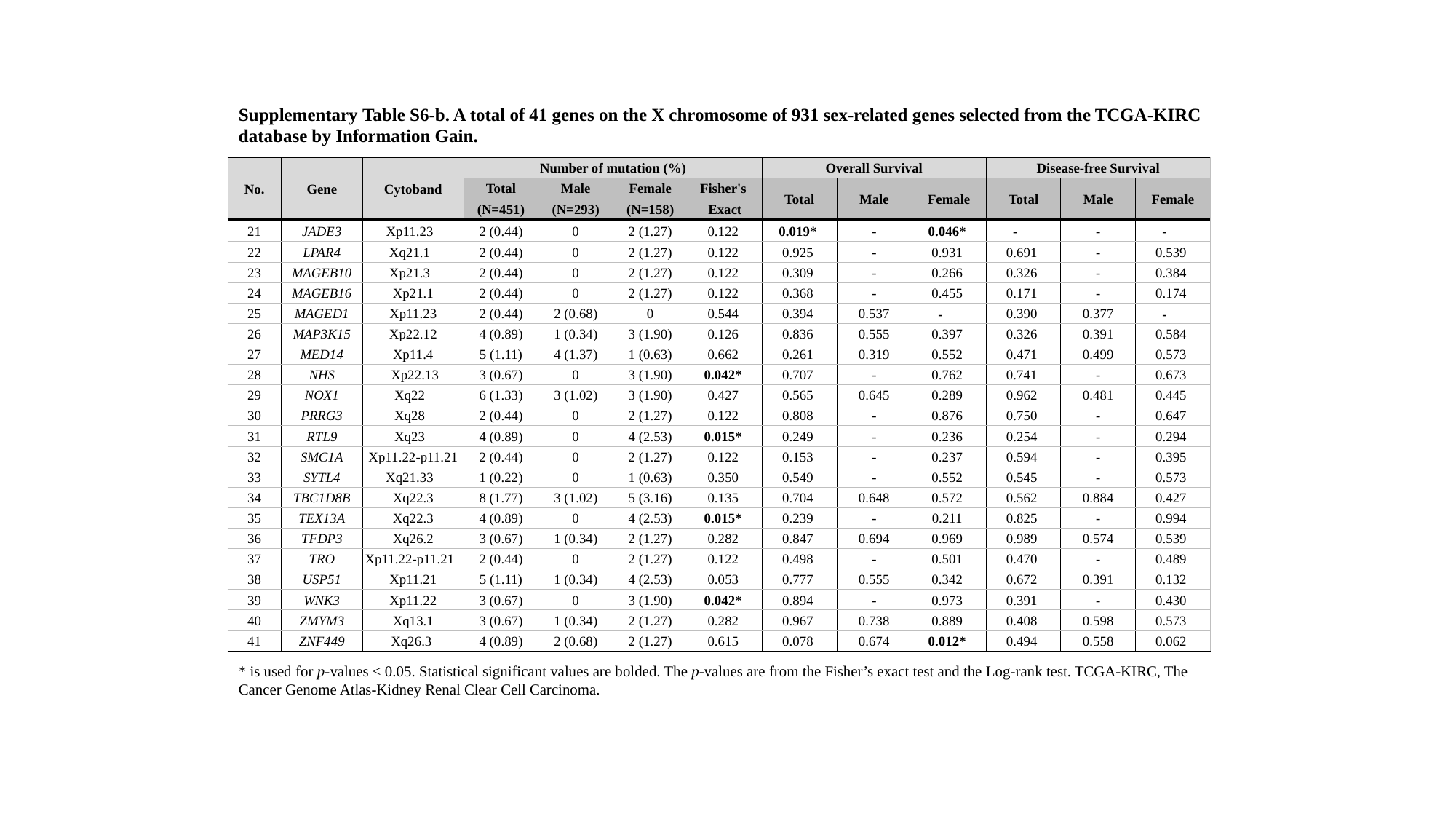

Supplementary Table S6-b. A total of 41 genes on the X chromosome of 931 sex-related genes selected from the TCGA-KIRC database by Information Gain.
| No. | Gene | Cytoband | Number of mutation (%) | | | | Overall Survival | | | Disease-free Survival | | |
| --- | --- | --- | --- | --- | --- | --- | --- | --- | --- | --- | --- | --- |
| | | | Total | Male | Female | Fisher's | Total | Male | Female | Total | Male | Female |
| | | | (N=451) | (N=293) | (N=158) | Exact | | | | | | |
| 21 | JADE3 | Xp11.23 | 2 (0.44) | 0 | 2 (1.27) | 0.122 | 0.019\* | - | 0.046\* | - | - | - |
| 22 | LPAR4 | Xq21.1 | 2 (0.44) | 0 | 2 (1.27) | 0.122 | 0.925 | - | 0.931 | 0.691 | - | 0.539 |
| 23 | MAGEB10 | Xp21.3 | 2 (0.44) | 0 | 2 (1.27) | 0.122 | 0.309 | - | 0.266 | 0.326 | - | 0.384 |
| 24 | MAGEB16 | Xp21.1 | 2 (0.44) | 0 | 2 (1.27) | 0.122 | 0.368 | - | 0.455 | 0.171 | - | 0.174 |
| 25 | MAGED1 | Xp11.23 | 2 (0.44) | 2 (0.68) | 0 | 0.544 | 0.394 | 0.537 | - | 0.390 | 0.377 | - |
| 26 | MAP3K15 | Xp22.12 | 4 (0.89) | 1 (0.34) | 3 (1.90) | 0.126 | 0.836 | 0.555 | 0.397 | 0.326 | 0.391 | 0.584 |
| 27 | MED14 | Xp11.4 | 5 (1.11) | 4 (1.37) | 1 (0.63) | 0.662 | 0.261 | 0.319 | 0.552 | 0.471 | 0.499 | 0.573 |
| 28 | NHS | Xp22.13 | 3 (0.67) | 0 | 3 (1.90) | 0.042\* | 0.707 | - | 0.762 | 0.741 | - | 0.673 |
| 29 | NOX1 | Xq22 | 6 (1.33) | 3 (1.02) | 3 (1.90) | 0.427 | 0.565 | 0.645 | 0.289 | 0.962 | 0.481 | 0.445 |
| 30 | PRRG3 | Xq28 | 2 (0.44) | 0 | 2 (1.27) | 0.122 | 0.808 | - | 0.876 | 0.750 | - | 0.647 |
| 31 | RTL9 | Xq23 | 4 (0.89) | 0 | 4 (2.53) | 0.015\* | 0.249 | - | 0.236 | 0.254 | - | 0.294 |
| 32 | SMC1A | Xp11.22-p11.21 | 2 (0.44) | 0 | 2 (1.27) | 0.122 | 0.153 | - | 0.237 | 0.594 | - | 0.395 |
| 33 | SYTL4 | Xq21.33 | 1 (0.22) | 0 | 1 (0.63) | 0.350 | 0.549 | - | 0.552 | 0.545 | - | 0.573 |
| 34 | TBC1D8B | Xq22.3 | 8 (1.77) | 3 (1.02) | 5 (3.16) | 0.135 | 0.704 | 0.648 | 0.572 | 0.562 | 0.884 | 0.427 |
| 35 | TEX13A | Xq22.3 | 4 (0.89) | 0 | 4 (2.53) | 0.015\* | 0.239 | - | 0.211 | 0.825 | - | 0.994 |
| 36 | TFDP3 | Xq26.2 | 3 (0.67) | 1 (0.34) | 2 (1.27) | 0.282 | 0.847 | 0.694 | 0.969 | 0.989 | 0.574 | 0.539 |
| 37 | TRO | Xp11.22-p11.21 | 2 (0.44) | 0 | 2 (1.27) | 0.122 | 0.498 | - | 0.501 | 0.470 | - | 0.489 |
| 38 | USP51 | Xp11.21 | 5 (1.11) | 1 (0.34) | 4 (2.53) | 0.053 | 0.777 | 0.555 | 0.342 | 0.672 | 0.391 | 0.132 |
| 39 | WNK3 | Xp11.22 | 3 (0.67) | 0 | 3 (1.90) | 0.042\* | 0.894 | - | 0.973 | 0.391 | - | 0.430 |
| 40 | ZMYM3 | Xq13.1 | 3 (0.67) | 1 (0.34) | 2 (1.27) | 0.282 | 0.967 | 0.738 | 0.889 | 0.408 | 0.598 | 0.573 |
| 41 | ZNF449 | Xq26.3 | 4 (0.89) | 2 (0.68) | 2 (1.27) | 0.615 | 0.078 | 0.674 | 0.012\* | 0.494 | 0.558 | 0.062 |
* is used for p-values < 0.05. Statistical significant values are bolded. The p-values are from the Fisher’s exact test and the Log-rank test. TCGA-KIRC, The Cancer Genome Atlas-Kidney Renal Clear Cell Carcinoma.

## Slide 8
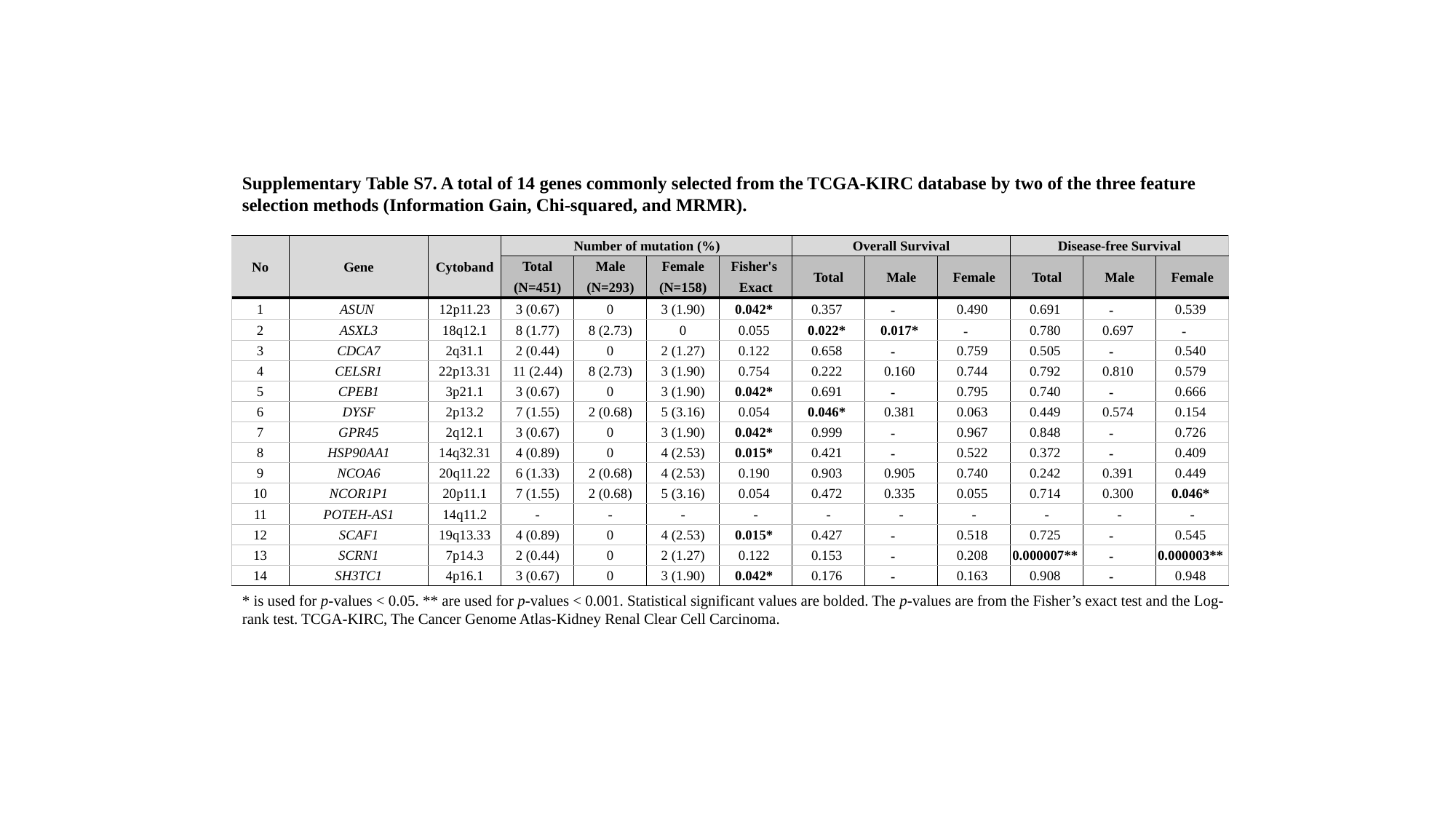

Supplementary Table S7. A total of 14 genes commonly selected from the TCGA-KIRC database by two of the three feature selection methods (Information Gain, Chi-squared, and MRMR).
| No | Gene | Cytoband | Number of mutation (%) | | | | Overall Survival | | | Disease-free Survival | | |
| --- | --- | --- | --- | --- | --- | --- | --- | --- | --- | --- | --- | --- |
| | | | Total | Male | Female | Fisher's | Total | Male | Female | Total | Male | Female |
| | | | (N=451) | (N=293) | (N=158) | Exact | | | | | | |
| 1 | ASUN | 12p11.23 | 3 (0.67) | 0 | 3 (1.90) | 0.042\* | 0.357 | - | 0.490 | 0.691 | - | 0.539 |
| 2 | ASXL3 | 18q12.1 | 8 (1.77) | 8 (2.73) | 0 | 0.055 | 0.022\* | 0.017\* | - | 0.780 | 0.697 | - |
| 3 | CDCA7 | 2q31.1 | 2 (0.44) | 0 | 2 (1.27) | 0.122 | 0.658 | - | 0.759 | 0.505 | - | 0.540 |
| 4 | CELSR1 | 22p13.31 | 11 (2.44) | 8 (2.73) | 3 (1.90) | 0.754 | 0.222 | 0.160 | 0.744 | 0.792 | 0.810 | 0.579 |
| 5 | CPEB1 | 3p21.1 | 3 (0.67) | 0 | 3 (1.90) | 0.042\* | 0.691 | - | 0.795 | 0.740 | - | 0.666 |
| 6 | DYSF | 2p13.2 | 7 (1.55) | 2 (0.68) | 5 (3.16) | 0.054 | 0.046\* | 0.381 | 0.063 | 0.449 | 0.574 | 0.154 |
| 7 | GPR45 | 2q12.1 | 3 (0.67) | 0 | 3 (1.90) | 0.042\* | 0.999 | - | 0.967 | 0.848 | - | 0.726 |
| 8 | HSP90AA1 | 14q32.31 | 4 (0.89) | 0 | 4 (2.53) | 0.015\* | 0.421 | - | 0.522 | 0.372 | - | 0.409 |
| 9 | NCOA6 | 20q11.22 | 6 (1.33) | 2 (0.68) | 4 (2.53) | 0.190 | 0.903 | 0.905 | 0.740 | 0.242 | 0.391 | 0.449 |
| 10 | NCOR1P1 | 20p11.1 | 7 (1.55) | 2 (0.68) | 5 (3.16) | 0.054 | 0.472 | 0.335 | 0.055 | 0.714 | 0.300 | 0.046\* |
| 11 | POTEH-AS1 | 14q11.2 | - | - | - | - | - | - | - | - | - | - |
| 12 | SCAF1 | 19q13.33 | 4 (0.89) | 0 | 4 (2.53) | 0.015\* | 0.427 | - | 0.518 | 0.725 | - | 0.545 |
| 13 | SCRN1 | 7p14.3 | 2 (0.44) | 0 | 2 (1.27) | 0.122 | 0.153 | - | 0.208 | 0.000007\*\* | - | 0.000003\*\* |
| 14 | SH3TC1 | 4p16.1 | 3 (0.67) | 0 | 3 (1.90) | 0.042\* | 0.176 | - | 0.163 | 0.908 | - | 0.948 |
* is used for p-values < 0.05. ** are used for p-values < 0.001. Statistical significant values are bolded. The p-values are from the Fisher’s exact test and the Log-rank test. TCGA-KIRC, The Cancer Genome Atlas-Kidney Renal Clear Cell Carcinoma.

## Slide 9
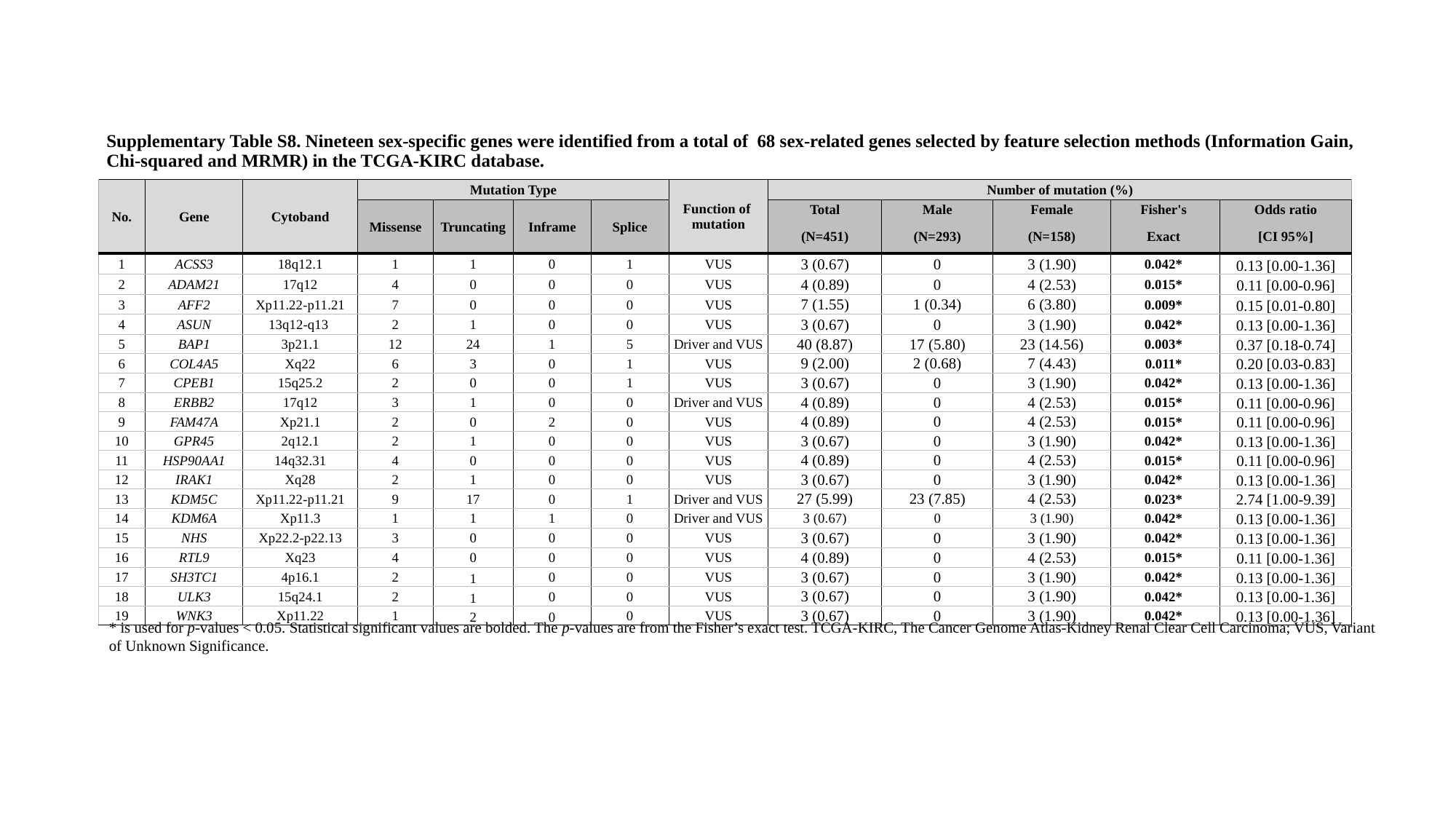

Supplementary Table S8. Nineteen sex-specific genes were identified from a total of 68 sex-related genes selected by feature selection methods (Information Gain, Chi-squared and MRMR) in the TCGA-KIRC database.
| No. | Gene | Cytoband | Mutation Type | | | | Function of mutation | Number of mutation (%) | | | | |
| --- | --- | --- | --- | --- | --- | --- | --- | --- | --- | --- | --- | --- |
| | | | Missense | Truncating | Inframe | Splice | | Total | Male | Female | Fisher's | Odds ratio |
| | | | | | | | | (N=451) | (N=293) | (N=158) | Exact | [CI 95%] |
| 1 | ACSS3 | 18q12.1 | 1 | 1 | 0 | 1 | VUS | 3 (0.67) | 0 | 3 (1.90) | 0.042\* | 0.13 [0.00-1.36] |
| 2 | ADAM21 | 17q12 | 4 | 0 | 0 | 0 | VUS | 4 (0.89) | 0 | 4 (2.53) | 0.015\* | 0.11 [0.00-0.96] |
| 3 | AFF2 | Xp11.22-p11.21 | 7 | 0 | 0 | 0 | VUS | 7 (1.55) | 1 (0.34) | 6 (3.80) | 0.009\* | 0.15 [0.01-0.80] |
| 4 | ASUN | 13q12-q13 | 2 | 1 | 0 | 0 | VUS | 3 (0.67) | 0 | 3 (1.90) | 0.042\* | 0.13 [0.00-1.36] |
| 5 | BAP1 | 3p21.1 | 12 | 24 | 1 | 5 | Driver and VUS | 40 (8.87) | 17 (5.80) | 23 (14.56) | 0.003\* | 0.37 [0.18-0.74] |
| 6 | COL4A5 | Xq22 | 6 | 3 | 0 | 1 | VUS | 9 (2.00) | 2 (0.68) | 7 (4.43) | 0.011\* | 0.20 [0.03-0.83] |
| 7 | CPEB1 | 15q25.2 | 2 | 0 | 0 | 1 | VUS | 3 (0.67) | 0 | 3 (1.90) | 0.042\* | 0.13 [0.00-1.36] |
| 8 | ERBB2 | 17q12 | 3 | 1 | 0 | 0 | Driver and VUS | 4 (0.89) | 0 | 4 (2.53) | 0.015\* | 0.11 [0.00-0.96] |
| 9 | FAM47A | Xp21.1 | 2 | 0 | 2 | 0 | VUS | 4 (0.89) | 0 | 4 (2.53) | 0.015\* | 0.11 [0.00-0.96] |
| 10 | GPR45 | 2q12.1 | 2 | 1 | 0 | 0 | VUS | 3 (0.67) | 0 | 3 (1.90) | 0.042\* | 0.13 [0.00-1.36] |
| 11 | HSP90AA1 | 14q32.31 | 4 | 0 | 0 | 0 | VUS | 4 (0.89) | 0 | 4 (2.53) | 0.015\* | 0.11 [0.00-0.96] |
| 12 | IRAK1 | Xq28 | 2 | 1 | 0 | 0 | VUS | 3 (0.67) | 0 | 3 (1.90) | 0.042\* | 0.13 [0.00-1.36] |
| 13 | KDM5C | Xp11.22-p11.21 | 9 | 17 | 0 | 1 | Driver and VUS | 27 (5.99) | 23 (7.85) | 4 (2.53) | 0.023\* | 2.74 [1.00-9.39] |
| 14 | KDM6A | Xp11.3 | 1 | 1 | 1 | 0 | Driver and VUS | 3 (0.67) | 0 | 3 (1.90) | 0.042\* | 0.13 [0.00-1.36] |
| 15 | NHS | Xp22.2-p22.13 | 3 | 0 | 0 | 0 | VUS | 3 (0.67) | 0 | 3 (1.90) | 0.042\* | 0.13 [0.00-1.36] |
| 16 | RTL9 | Xq23 | 4 | 0 | 0 | 0 | VUS | 4 (0.89) | 0 | 4 (2.53) | 0.015\* | 0.11 [0.00-1.36] |
| 17 | SH3TC1 | 4p16.1 | 2 | 1 | 0 | 0 | VUS | 3 (0.67) | 0 | 3 (1.90) | 0.042\* | 0.13 [0.00-1.36] |
| 18 | ULK3 | 15q24.1 | 2 | 1 | 0 | 0 | VUS | 3 (0.67) | 0 | 3 (1.90) | 0.042\* | 0.13 [0.00-1.36] |
| 19 | WNK3 | Xp11.22 | 1 | 2 | 0 | 0 | VUS | 3 (0.67) | 0 | 3 (1.90) | 0.042\* | 0.13 [0.00-1.36] |
* is used for p-values < 0.05. Statistical significant values are bolded. The p-values are from the Fisher’s exact test. TCGA-KIRC, The Cancer Genome Atlas-Kidney Renal Clear Cell Carcinoma; VUS, Variant of Unknown Significance.

## Slide 10
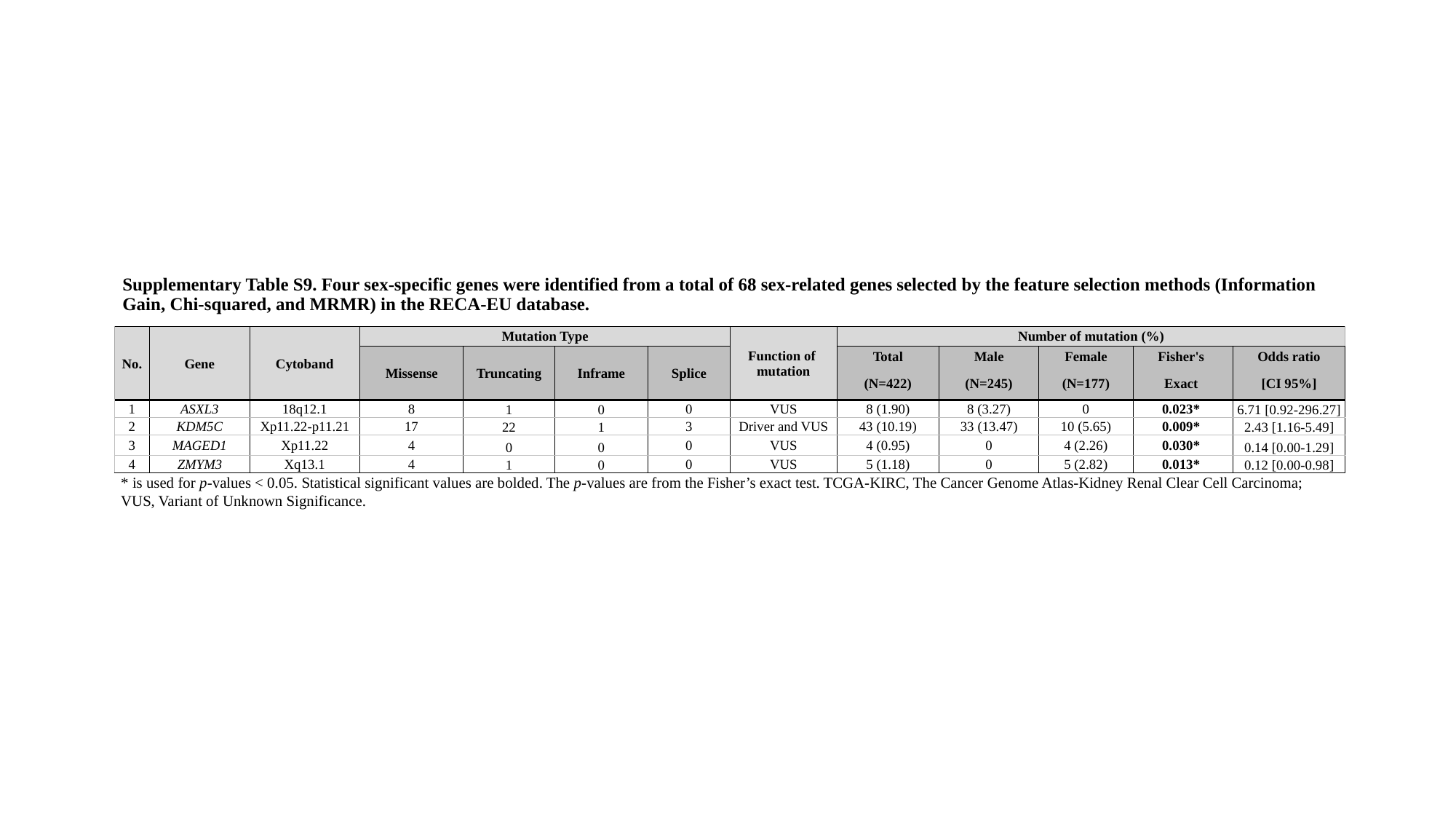

Supplementary Table S9. Four sex-specific genes were identified from a total of 68 sex-related genes selected by the feature selection methods (Information Gain, Chi-squared, and MRMR) in the RECA-EU database.
| No. | Gene | Cytoband | Mutation Type | | | | Function of mutation | Number of mutation (%) | | | | |
| --- | --- | --- | --- | --- | --- | --- | --- | --- | --- | --- | --- | --- |
| | | | Missense | Truncating | Inframe | Splice | | Total | Male | Female | Fisher's | Odds ratio |
| | | | | | | | | (N=422) | (N=245) | (N=177) | Exact | [CI 95%] |
| 1 | ASXL3 | 18q12.1 | 8 | 1 | 0 | 0 | VUS | 8 (1.90) | 8 (3.27) | 0 | 0.023\* | 6.71 [0.92-296.27] |
| 2 | KDM5C | Xp11.22-p11.21 | 17 | 22 | 1 | 3 | Driver and VUS | 43 (10.19) | 33 (13.47) | 10 (5.65) | 0.009\* | 2.43 [1.16-5.49] |
| 3 | MAGED1 | Xp11.22 | 4 | 0 | 0 | 0 | VUS | 4 (0.95) | 0 | 4 (2.26) | 0.030\* | 0.14 [0.00-1.29] |
| 4 | ZMYM3 | Xq13.1 | 4 | 1 | 0 | 0 | VUS | 5 (1.18) | 0 | 5 (2.82) | 0.013\* | 0.12 [0.00-0.98] |
* is used for p-values < 0.05. Statistical significant values are bolded. The p-values are from the Fisher’s exact test. TCGA-KIRC, The Cancer Genome Atlas-Kidney Renal Clear Cell Carcinoma; VUS, Variant of Unknown Significance.

## Slide 11
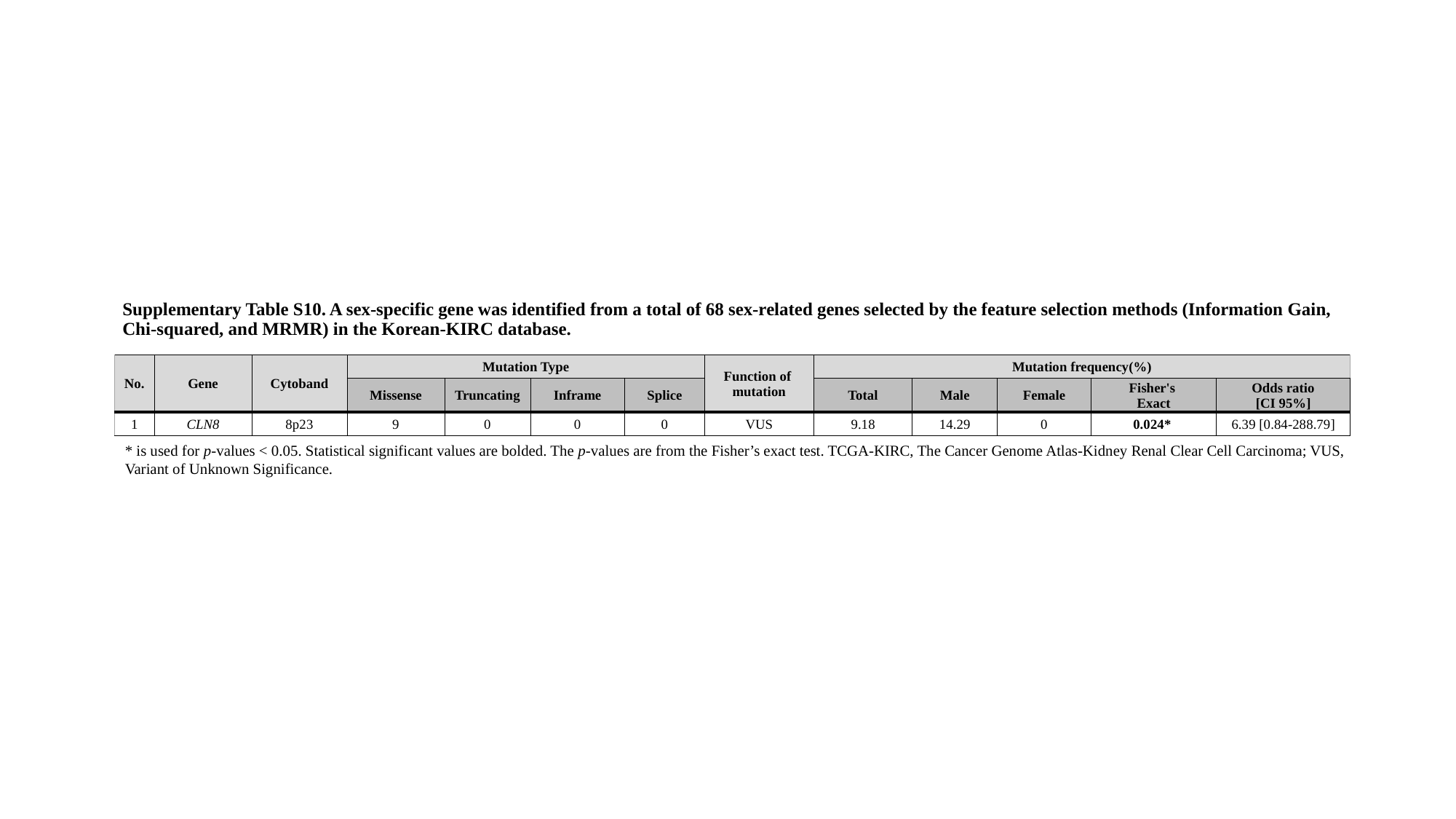

Supplementary Table S10. A sex-specific gene was identified from a total of 68 sex-related genes selected by the feature selection methods (Information Gain, Chi-squared, and MRMR) in the Korean-KIRC database.
| No. | Gene | Cytoband | Mutation Type | | | | Function of mutation | Mutation frequency(%) | | | | |
| --- | --- | --- | --- | --- | --- | --- | --- | --- | --- | --- | --- | --- |
| | | | Missense | Truncating | Inframe | Splice | | Total | Male | Female | Fisher's Exact | Odds ratio [CI 95%] |
| 1 | CLN8 | 8p23 | 9 | 0 | 0 | 0 | VUS | 9.18 | 14.29 | 0 | 0.024\* | 6.39 [0.84-288.79] |
* is used for p-values < 0.05. Statistical significant values are bolded. The p-values are from the Fisher’s exact test. TCGA-KIRC, The Cancer Genome Atlas-Kidney Renal Clear Cell Carcinoma; VUS, Variant of Unknown Significance.

## Slide 12
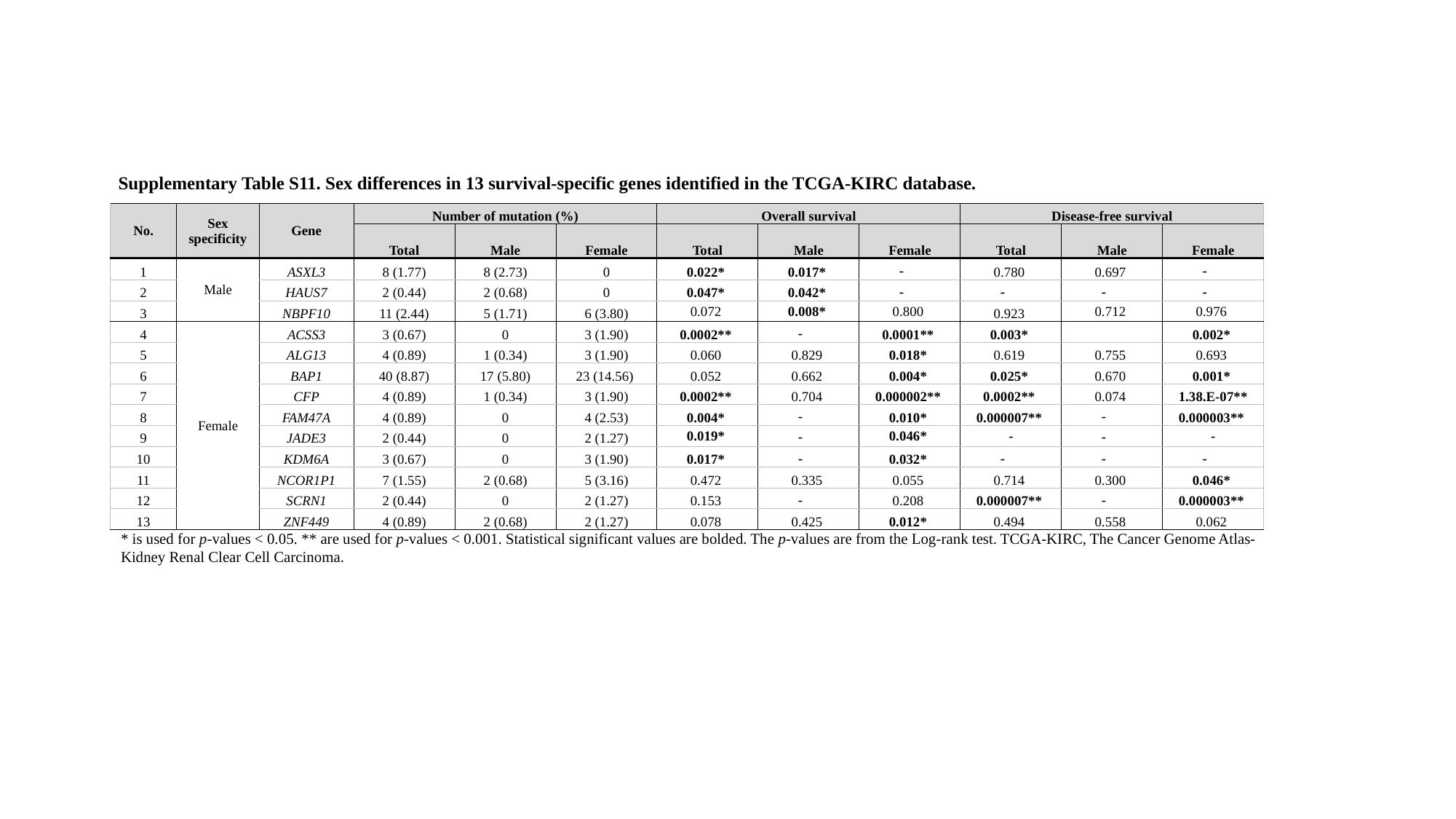

Supplementary Table S11. Sex differences in 13 survival-specific genes identified in the TCGA-KIRC database.
| No. | Sex specificity | Gene | Number of mutation (%) | | | Overall survival | | | Disease-free survival | | |
| --- | --- | --- | --- | --- | --- | --- | --- | --- | --- | --- | --- |
| | | | Total | Male | Female | Total | Male | Female | Total | Male | Female |
| 1 | Male | ASXL3 | 8 (1.77) | 8 (2.73) | 0 | 0.022\* | 0.017\* | - | 0.780 | 0.697 | - |
| 2 | | HAUS7 | 2 (0.44) | 2 (0.68) | 0 | 0.047\* | 0.042\* | - | - | - | - |
| 3 | | NBPF10 | 11 (2.44) | 5 (1.71) | 6 (3.80) | 0.072 | 0.008\* | 0.800 | 0.923 | 0.712 | 0.976 |
| 4 | Female | ACSS3 | 3 (0.67) | 0 | 3 (1.90) | 0.0002\*\* | - | 0.0001\*\* | 0.003\* | | 0.002\* |
| 5 | | ALG13 | 4 (0.89) | 1 (0.34) | 3 (1.90) | 0.060 | 0.829 | 0.018\* | 0.619 | 0.755 | 0.693 |
| 6 | | BAP1 | 40 (8.87) | 17 (5.80) | 23 (14.56) | 0.052 | 0.662 | 0.004\* | 0.025\* | 0.670 | 0.001\* |
| 7 | | CFP | 4 (0.89) | 1 (0.34) | 3 (1.90) | 0.0002\*\* | 0.704 | 0.000002\*\* | 0.0002\*\* | 0.074 | 1.38.E-07\*\* |
| 8 | | FAM47A | 4 (0.89) | 0 | 4 (2.53) | 0.004\* | - | 0.010\* | 0.000007\*\* | - | 0.000003\*\* |
| 9 | | JADE3 | 2 (0.44) | 0 | 2 (1.27) | 0.019\* | - | 0.046\* | - | - | - |
| 10 | | KDM6A | 3 (0.67) | 0 | 3 (1.90) | 0.017\* | - | 0.032\* | - | - | - |
| 11 | | NCOR1P1 | 7 (1.55) | 2 (0.68) | 5 (3.16) | 0.472 | 0.335 | 0.055 | 0.714 | 0.300 | 0.046\* |
| 12 | | SCRN1 | 2 (0.44) | 0 | 2 (1.27) | 0.153 | - | 0.208 | 0.000007\*\* | - | 0.000003\*\* |
| 13 | | ZNF449 | 4 (0.89) | 2 (0.68) | 2 (1.27) | 0.078 | 0.425 | 0.012\* | 0.494 | 0.558 | 0.062 |
* is used for p-values < 0.05. ** are used for p-values < 0.001. Statistical significant values are bolded. The p-values are from the Log-rank test. TCGA-KIRC, The Cancer Genome Atlas-Kidney Renal Clear Cell Carcinoma.

## Slide 13
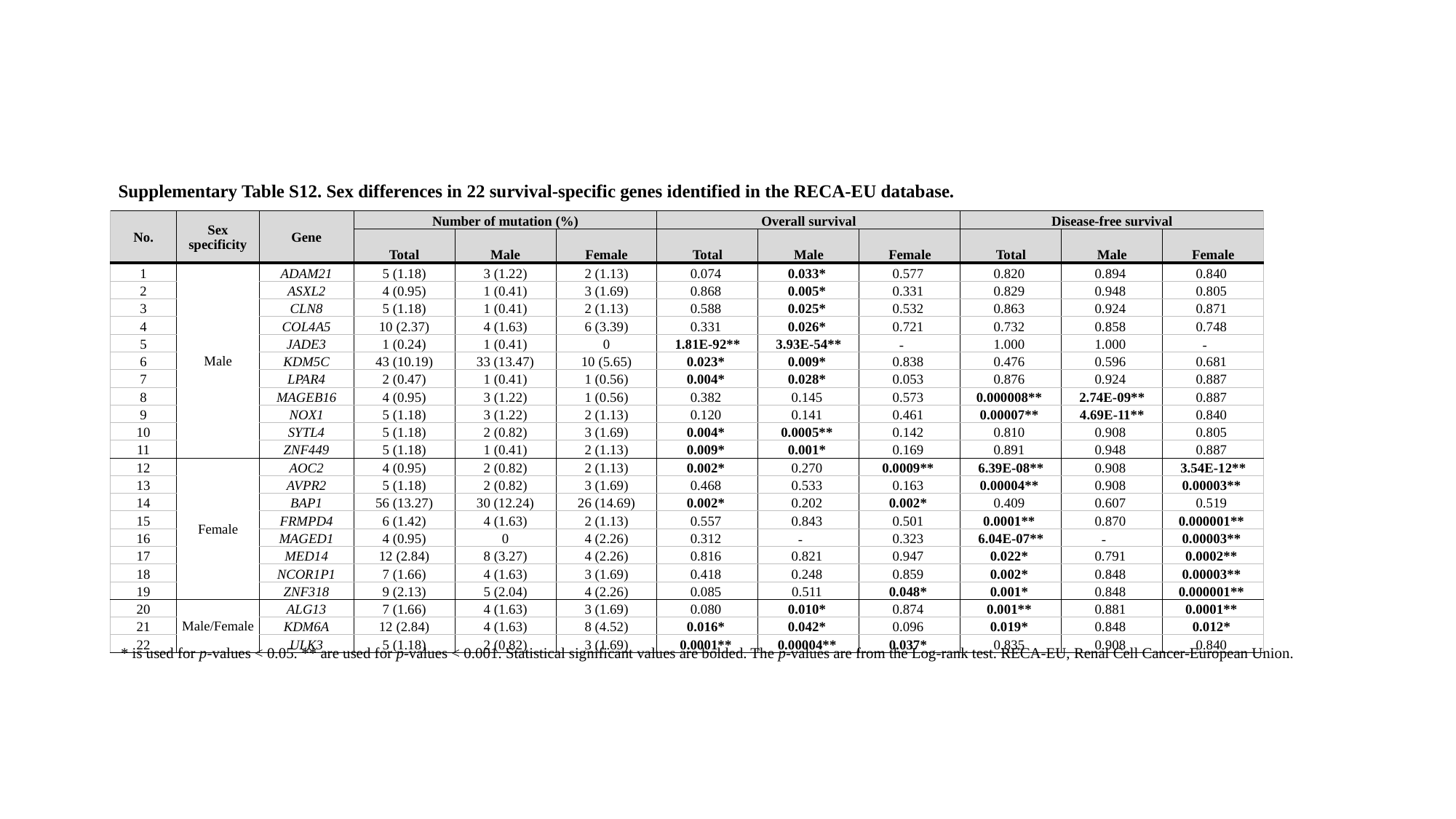

Supplementary Table S12. Sex differences in 22 survival-specific genes identified in the RECA-EU database.
| No. | Sex specificity | Gene | Number of mutation (%) | | | Overall survival | | | Disease-free survival | | |
| --- | --- | --- | --- | --- | --- | --- | --- | --- | --- | --- | --- |
| | | | Total | Male | Female | Total | Male | Female | Total | Male | Female |
| 1 | Male | ADAM21 | 5 (1.18) | 3 (1.22) | 2 (1.13) | 0.074 | 0.033\* | 0.577 | 0.820 | 0.894 | 0.840 |
| 2 | | ASXL2 | 4 (0.95) | 1 (0.41) | 3 (1.69) | 0.868 | 0.005\* | 0.331 | 0.829 | 0.948 | 0.805 |
| 3 | | CLN8 | 5 (1.18) | 1 (0.41) | 2 (1.13) | 0.588 | 0.025\* | 0.532 | 0.863 | 0.924 | 0.871 |
| 4 | | COL4A5 | 10 (2.37) | 4 (1.63) | 6 (3.39) | 0.331 | 0.026\* | 0.721 | 0.732 | 0.858 | 0.748 |
| 5 | | JADE3 | 1 (0.24) | 1 (0.41) | 0 | 1.81E-92\*\* | 3.93E-54\*\* | - | 1.000 | 1.000 | - |
| 6 | | KDM5C | 43 (10.19) | 33 (13.47) | 10 (5.65) | 0.023\* | 0.009\* | 0.838 | 0.476 | 0.596 | 0.681 |
| 7 | | LPAR4 | 2 (0.47) | 1 (0.41) | 1 (0.56) | 0.004\* | 0.028\* | 0.053 | 0.876 | 0.924 | 0.887 |
| 8 | | MAGEB16 | 4 (0.95) | 3 (1.22) | 1 (0.56) | 0.382 | 0.145 | 0.573 | 0.000008\*\* | 2.74E-09\*\* | 0.887 |
| 9 | | NOX1 | 5 (1.18) | 3 (1.22) | 2 (1.13) | 0.120 | 0.141 | 0.461 | 0.00007\*\* | 4.69E-11\*\* | 0.840 |
| 10 | | SYTL4 | 5 (1.18) | 2 (0.82) | 3 (1.69) | 0.004\* | 0.0005\*\* | 0.142 | 0.810 | 0.908 | 0.805 |
| 11 | | ZNF449 | 5 (1.18) | 1 (0.41) | 2 (1.13) | 0.009\* | 0.001\* | 0.169 | 0.891 | 0.948 | 0.887 |
| 12 | Female | AOC2 | 4 (0.95) | 2 (0.82) | 2 (1.13) | 0.002\* | 0.270 | 0.0009\*\* | 6.39E-08\*\* | 0.908 | 3.54E-12\*\* |
| 13 | | AVPR2 | 5 (1.18) | 2 (0.82) | 3 (1.69) | 0.468 | 0.533 | 0.163 | 0.00004\*\* | 0.908 | 0.00003\*\* |
| 14 | | BAP1 | 56 (13.27) | 30 (12.24) | 26 (14.69) | 0.002\* | 0.202 | 0.002\* | 0.409 | 0.607 | 0.519 |
| 15 | | FRMPD4 | 6 (1.42) | 4 (1.63) | 2 (1.13) | 0.557 | 0.843 | 0.501 | 0.0001\*\* | 0.870 | 0.000001\*\* |
| 16 | | MAGED1 | 4 (0.95) | 0 | 4 (2.26) | 0.312 | - | 0.323 | 6.04E-07\*\* | - | 0.00003\*\* |
| 17 | | MED14 | 12 (2.84) | 8 (3.27) | 4 (2.26) | 0.816 | 0.821 | 0.947 | 0.022\* | 0.791 | 0.0002\*\* |
| 18 | | NCOR1P1 | 7 (1.66) | 4 (1.63) | 3 (1.69) | 0.418 | 0.248 | 0.859 | 0.002\* | 0.848 | 0.00003\*\* |
| 19 | | ZNF318 | 9 (2.13) | 5 (2.04) | 4 (2.26) | 0.085 | 0.511 | 0.048\* | 0.001\* | 0.848 | 0.000001\*\* |
| 20 | Male/Female | ALG13 | 7 (1.66) | 4 (1.63) | 3 (1.69) | 0.080 | 0.010\* | 0.874 | 0.001\*\* | 0.881 | 0.0001\*\* |
| 21 | | KDM6A | 12 (2.84) | 4 (1.63) | 8 (4.52) | 0.016\* | 0.042\* | 0.096 | 0.019\* | 0.848 | 0.012\* |
| 22 | | ULK3 | 5 (1.18) | 2 (0.82) | 3 (1.69) | 0.0001\*\* | 0.00004\*\* | 0.037\* | 0.835 | 0.908 | 0.840 |
* is used for p-values < 0.05. ** are used for p-values < 0.001. Statistical significant values are bolded. The p-values are from the Log-rank test. RECA-EU, Renal Cell Cancer-European Union.

## Slide 14
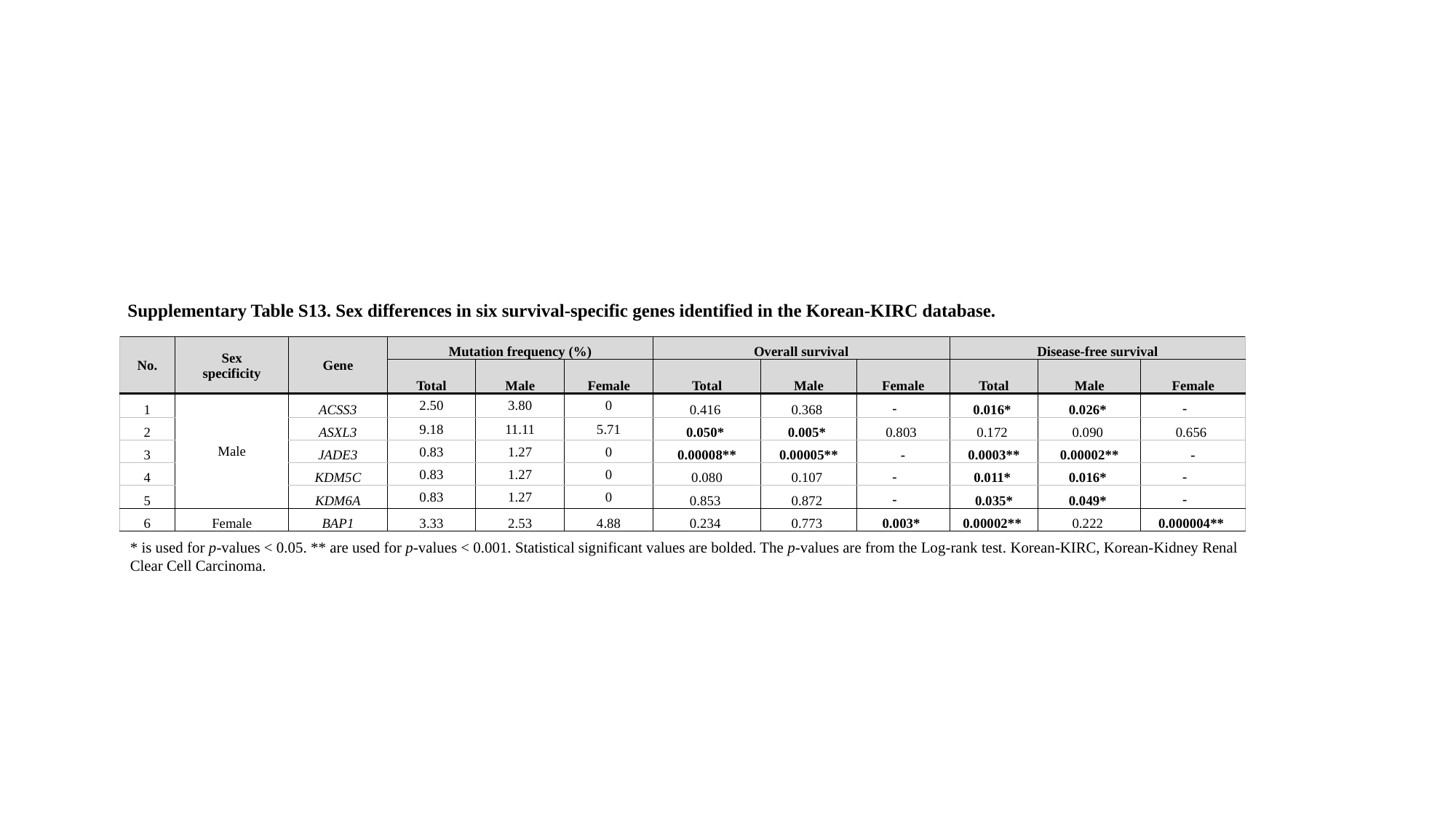

Supplementary Table S13. Sex differences in six survival-specific genes identified in the Korean-KIRC database.
| No. | Sex specificity | Gene | Mutation frequency (%) | | | Overall survival | | | Disease-free survival | | |
| --- | --- | --- | --- | --- | --- | --- | --- | --- | --- | --- | --- |
| | | | Total | Male | Female | Total | Male | Female | Total | Male | Female |
| 1 | Male | ACSS3 | 2.50 | 3.80 | 0 | 0.416 | 0.368 | - | 0.016\* | 0.026\* | - |
| 2 | | ASXL3 | 9.18 | 11.11 | 5.71 | 0.050\* | 0.005\* | 0.803 | 0.172 | 0.090 | 0.656 |
| 3 | | JADE3 | 0.83 | 1.27 | 0 | 0.00008\*\* | 0.00005\*\* | - | 0.0003\*\* | 0.00002\*\* | - |
| 4 | | KDM5C | 0.83 | 1.27 | 0 | 0.080 | 0.107 | - | 0.011\* | 0.016\* | - |
| 5 | | KDM6A | 0.83 | 1.27 | 0 | 0.853 | 0.872 | - | 0.035\* | 0.049\* | - |
| 6 | Female | BAP1 | 3.33 | 2.53 | 4.88 | 0.234 | 0.773 | 0.003\* | 0.00002\*\* | 0.222 | 0.000004\*\* |
* is used for p-values < 0.05. ** are used for p-values < 0.001. Statistical significant values are bolded. The p-values are from the Log-rank test. Korean-KIRC, Korean-Kidney Renal Clear Cell Carcinoma.

## Slide 15
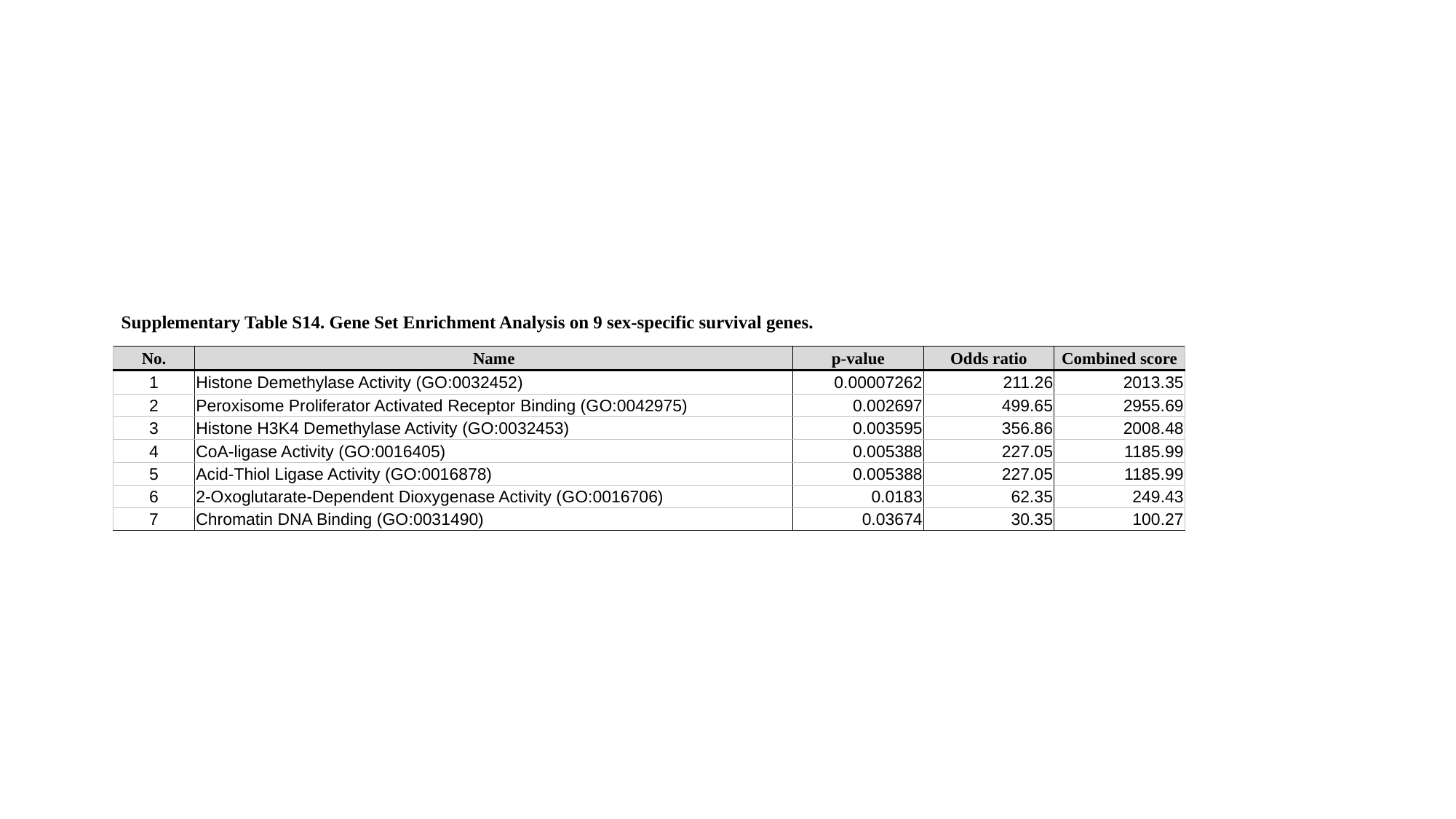

Supplementary Table S14. Gene Set Enrichment Analysis on 9 sex-specific survival genes.
| No. | Name | p-value | Odds ratio | Combined score |
| --- | --- | --- | --- | --- |
| 1 | Histone Demethylase Activity (GO:0032452) | 0.00007262 | 211.26 | 2013.35 |
| 2 | Peroxisome Proliferator Activated Receptor Binding (GO:0042975) | 0.002697 | 499.65 | 2955.69 |
| 3 | Histone H3K4 Demethylase Activity (GO:0032453) | 0.003595 | 356.86 | 2008.48 |
| 4 | CoA-ligase Activity (GO:0016405) | 0.005388 | 227.05 | 1185.99 |
| 5 | Acid-Thiol Ligase Activity (GO:0016878) | 0.005388 | 227.05 | 1185.99 |
| 6 | 2-Oxoglutarate-Dependent Dioxygenase Activity (GO:0016706) | 0.0183 | 62.35 | 249.43 |
| 7 | Chromatin DNA Binding (GO:0031490) | 0.03674 | 30.35 | 100.27 |
